# Dynamic Application of High and Low Red:Blue Ratios During Lettuce Development Shifts Growth and Metabolite Allocation

**DOI:** 10.1111/ppl.70456

**Published:** 2025-08-13

**Authors:** Jordan B. Van Brenk, Lonneke Hendriks, Andrea Rei, Leo F. M. Marcelis, Julian C. Verdonk

**Affiliations:** ^1^ Horticulture and Product Physiology Wageningen University and Research, Plant Sciences Group Wageningen the Netherlands

**Keywords:** controlled environment agriculture, dynamic lighting, lettuce, light quality, phenylpropanoids, product quality, specialized metabolites

## Abstract

Plants respond to light spectrum in different ways. When using red:blue (R:B) lighting during cultivation, high R:B can increase plant growth and carbohydrates. Conversely, low R:B can increase phytochemicals and nutritional compounds. The aim of this experiment was to utilize dynamic R:B spectra to define this interplay between growth and phytochemical production by determining if spectral effects on growth are due to preferential carbon allocation to different phenylpropanoids. Here, lettuce ( *Lactuca sativa* L.) was grown under dynamic applications of high R:B (R:B_89:11_) or low R:B (R:B_50:50_) for three 9‐day phases. Plant growth decreased based on cumulative exposure to low R:B. Two groups of phenylpropanoids, flavonoids and anthocyanins, were induced under low R:B, with similar concentrations regardless of whether plants were exposed to low R:B for the final 9 days or all 27 days. Red lettuce preferentially produced anthocyanins over quercetin, whereas green lettuce maintained high quercetin. However, lignin, structural phenylpropanoids, and cell wall‐associated compounds were unaffected by R:B. Growth effects from R:B were instead linked to available energy from carbohydrates, which increased under high R:B. In conclusion, phytochemical content depends on the spectral conditions that plants receive during the final growth phase, while growth depends on the cumulative spectra. Using dynamic light application can therefore balance growth and phenylpropanoids.

## 1 Introduction

Due to an increased demand for nutritional foods, there is a drive to improve upon existing systems or develop new agricultural techniques. Among these newer techniques is vertical farming, a system of controlled environment agriculture wherein plants are grown indoors in vertically stacked growth areas that are artificially illuminated (van Delden et al. 2021). In vertical farming, crops can be grown year‐round, in any area with electricity, while reducing the use of water, pesticides, land area, and transportation (Benke and Tomkins 2017; van Delden et al. 2021). Lettuce ( *Lactuca sativa* L.) is often grown due to its short growth cycle, small stature, and consistent market demand (Touliatos et al. 2016). Lettuce has been suggested to have many health benefits as it is a source of several health compounds, such as carbohydrates, ascorbic acid, and phenylpropanoids (Fasciolo et al. 2024; Kim et al. 2016; Williams et al. 2004). During cultivation, if the content of these nutritional compounds is increased, this improves crop quality. Additionally, increased carbohydrates and vitamin C at harvest have been correlated with extended shelf life (Lee et al. 2017; Min et al. 2021).

In vertical farms, red and blue light‐emitting diodes (LEDs) are often used as light sources, as these wavelengths are efficient for plant photosynthesis (Massa et al. 2008; McCree 1971; Naznin et al. 2019). Red light (R; 600–700 nm) is commonly used in high fractions as R LEDs have high electrical efficacy (conversion efficiency of electricity into light) and can produce high crop growth (Azad et al. 2020; Kusuma et al. 2020; Son and Oh 2013). However, using only R may cause serious growth abnormalities (Trouwborst et al. 2016), which is alleviated by adding blue light (B; 400–500 nm), in a combined red:blue (R:B) spectrum (Miao et al. 2019). B is also important for photosynthesis and can increase phytochemical content (Liu and van Iersel 2021). However, high B fraction may reduce crop growth (Clavijo‐Herrera et al. 2018; Kong and Nemali 2021; Snowden et al. 2016); blue LEDs also have lower efficacy, and a blue LED therefore uses more electrical energy to produce the same number of photons as a red LED (Kusuma et al. 2022). Hence, light conditions should be customized during plant growth to improve plants' growth and even their nutritional quality (Kaiser et al. 2024). This can be made possible via dynamic application of R:B spectra, which has the potential to synergistically benefit plant growth, nutritional quality, and electrical energy consumption in plant production (Chen, Li, et al. 2021; Kaiser et al. 2024).

Although low R:B (high B fraction) can hamper plant growth, it can also improve phytochemical and nutritional content in several plant species (Fasciolo et al. 2024; Kong and Nemali 2021; Samuolienė et al. 2013; Van Brenk et al. 2024, 2025). These responses are often mediated via light recognition by photoreceptors such as R‐responding phytochromes (Neff and Chory 1998; Sharrock 2008) and B‐responding cryptochromes and phototropins (Christie 2007; Neff and Chory 1998; Tissot and Ulm 2020; Yu et al. 2010). For example, B activates cryptochrome, which (among a host of other responses) reduces the ability of CONSTITUTIVELY PHOTOMORPHOGENIC 1 (COP1) to degrade the transcription factor ELONGATED HYPOCOTYL 5 (HY5) (Gangappa and Botto 2016; Ma et al. 2021; Tissot and Ulm 2020; Yu et al. 2010). The HY5 transcription factor is crucial for light‐mediated regulation of many metabolic pathways that affect growth and specialized metabolite production (Gangappa and Botto 2016; Ma et al. 2021; Tissot and Ulm 2020; Yu et al. 2010). Through responses such as these, low R:B can enhance the production of several nutritionally relevant compounds, including chlorophyll, carotenoids, and some phenylpropanoids (Dixon and Paiva 1995; Li et al. 2021; Naznin et al. 2019; Van Brenk et al. 2024). Phenylpropanoids are a diverse group of phytochemicals synthesized from the aromatic amino acid phenylalanine (Vogt 2009). They have various ecological functions, including their antioxidant capacity (anthocyanins/flavonoids), ability to filter light (anthocyanins), and structural contributions (lignin) (Tissier et al. 2015; Vogt 2009). Flavonoids—a large group of phenylpropanoids—are involved in plant defense and can protect from reactive oxygen species (ROS) by scavenging ROS via their antioxidant capacity (Pietta 2000). By scavenging ROS, flavonoids maintain plant health and growth under conditions that can induce ROS, such as light environments with low R:B (Pietta 2000; Son and Oh 2013). Anthocyanins, a subclass of flavonoids, also increase with low R:B. They filter high‐energy light (such as B light or high light intensity) before it can negatively impact photosynthetic machinery (Naing and Kim 2021). Their light‐filtering role in addition to their antioxidant capacity as flavonoids protect plants from many potential sources of stress (Gould 2004; Kovinich et al. 2015; Naing and Kim 2021). Notably, anthocyanins are found primarily in red lettuce and in low concentrations or not at all in green lettuce, as anthocyanins produce red, purple, and blue pigments (Mulabagal et al. 2010). The antioxidant traits of these phenylpropanoids can improve crop quality, nutritional value, and consumer preference (Franco Lucas et al. 2021; Khoo et al. 2017; Li et al. 2017).

Another important phenylpropanoid is lignin, a complex structural phenolic polymer that contributes to plant growth (Xie et al. 2018). Lignin facilitates growth as a crucial component in the secondary cell wall, providing rigidity and structure (Boerjan et al. 2003). It facilitates water and mineral transport as part of vascular bundle formation (Liu et al. 2018; Xie et al. 2018), and has roles in plant pathogen defense (Vanholme et al. 2012; Xie et al. 2018). Because of its ubiquitous presence in plants, lignin is one of the most abundant biological polymers on the planet (Boerjan et al. 2003; Vogt 2009). Notably, lignin and flavonoid synthesis share metabolic precursors, and therefore are co‐dependent on the same resources (Shi et al. 2022), such as carbon from primary metabolites, including carbohydrates (Creasy 1968). As a theoretical example, if the carbon source from their shared precursors is stable (and finite), then an increase in carbon flux toward flavonoids may result in reduced carbon available for lignin, due to their inherent competition for the same resources. Some contrasting data exist on the balance between these compounds. Besseau et al. (2007) showed increased flavonoid production in lignin‐repressed plants, while Li et al. (2010) found that lignin responses may be independent of flavonoids. Despite lignin's contribution to plant growth and structure, the effects of light on lignin content are seldom described, except for some lignin studies in plants grown under foliar shade conditions (Hussain et al. 2019; Ranade et al. 2022; Wu et al. 2017).

The trade‐offs between growth and quality are important considerations for plants cultivated under different light spectra. We hypothesized that the resource allocation toward flavonoid biosynthesis under low R:B may reduce the available resources for lignin production, which may be the cause of reduced plant growth. Additionally, low R:B applied at the end of cultivation may lead to a higher phytochemical content at harvest, which is important for the nutritional value of edible plants. The aim of this experiment was to utilize dynamic R:B spectra to define this interplay between growth (by analyzing morphology and lignin content) and quality (by analyzing pigments and flavonoids), considering the plants' accessible energy (in the form of carbohydrates). We sought to determine if this interplay is due to preferential carbon allocation to different phenylpropanoids. In this study, a high R:B ratio (R:B_89:11_) and a low ratio (R:B_50:50_) were applied dynamically at different growth phases of two lettuce cultivars, a red‐leafed variety and a green‐leafed variety. This was performed by exposing plants to one of the two R:B ratios for three 9‐day phases, in each combination of R:B within each phase. Morphological and metabolic analysis were performed at the beginning and end of each growth phase to quantify the growth and quality effects of these dynamic R:B combinations. This unique phase‐based experimental design facilitated the determination of the interplay between growth and quality under different R:B spectra. Our dynamic R:B applications displayed how plants are affected by the total exposure to high or low R:B, shorter‐term exposures to high or low R:B, and the impact of the timing of R:B exposure.

## 2 Materials and Methods

### 2.1 Experimental Setup

#### 2.1.1 Sowing and Germination

Seeds of two multi‐leaf lettuce cultivars ( *Lactuca sativa* L. cv. “Greenflash” and *Lactuca sativa* L. cv. “Redflash”; Nunhems BV, Nunhem, the Netherlands) were sown in individual rockwool plugs (Grodan), covered with a layer of vermiculite, placed in plastic trays containing tap water, and covered with a transparent plastic lid to maintain humidity. After sowing, the seeds in the covered trays were stratified for 3 days in the dark (4°C). Following stratification, the transparent‐lidded trays with seeds were moved to germinate for 9 days in a climate chamber. Light conditions were an 18 h photoperiod at 130 μmol m^−2^ s^−1^ PAR with a red:blue ratio of 89:11 (R:B_89:11_), with a red peak of ~655 nm and a blue peak of ~450 nm provided by Greenpower Dynamic LED modules (GPL PM 168 DRBWFR L120 G3.0 C4 N4; Signify), and a 6 h night period. For better light distribution, F‐clean ETFE film (AGC Chemicals Europe Commercial Centre) was suspended beneath the LEDs. The chamber was maintained at 23°C during the day and 21°C at night, at 70% relative humidity, and an ambient CO_2_. After 9 days of germination, the seedlings in plugs were transplanted to rockwool blocks (7.5 × 7.5 × 6.5 cm, L × W × H; Grodan). These rockwool blocks were soaked in tap water before transplanting. Following transplant, rockwool blocks with seedlings were placed in individual compartments in the same climate chamber, which were separated by white plastic to avoid light contamination. Climate chamber conditions after transplanting were the same as germination, except for the experimental light treatments. Greenflash and Redflash were arranged in alternating rows at a density of 73 plants m^−2^. Plants were irrigated using an ebb‐and‐flood system with the same nutrient solution (EC = 2.3 dS m^−1^; pH = 6–6.5) described in Van Brenk et al. (2024).

#### 2.1.2 Light Treatments

**FIGURE 1:**
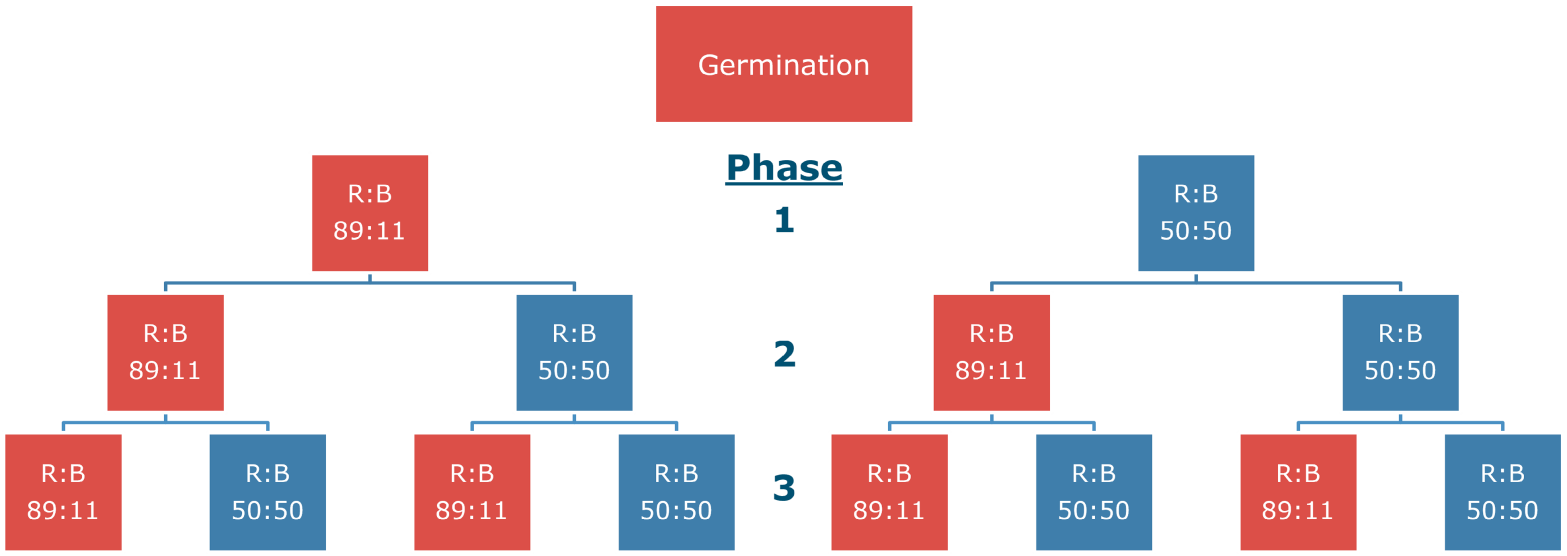
Experimental setup of light treatments. The experiment was divided into three phases of growth that lasted 9 days each, for a total growth duration of 27 days after transplanting seedlings (germination). Plants were split up for each phase to one of two different spectral compositions, a red:blue ratio of R:B_89:11_ (red boxes) or R:B_50:50_ (blue boxes). This expanded for each growth phase, as plants that received either R:B_89:11_ or R:B_50:50_ in the previous phase still received either R:B_89:11_ or R:B_50:50_ during the new phase. Plants were harvested after each phase for measurements.

Following transplant, the experiment was divided into three phases of growth that lasted 9 days each, for a total growth duration of 27 days after transplant (DAT). During each phase, plants were grown under one of two spectral compositions, either a red:blue ratio (R:B) of R:B_89:11_ (high R:B) or R:B_50:50_ (low R:B) (Figure 1). This was performed for each phase, compounding on the previous phases' treatments. Consequently, at the end of each phase of growth, the number of treatments doubled. This resulted in two treatments after phase 1 (R:B_89:11_ and R:B_50:50_), four treatments after phase two (R:B_89:11➔89:11_; R:B_89:11➔50:50_; R:B_50:50➔89:11_; and R:B_50:50➔50:50_), and eight treatments after phase 3 (R:B_89:11➔89:11➔89:11_; R:B_89:11➔89:11➔50:50_; R:B_89:11➔50:50➔89:11_; R:B_89:11➔50:50➔50:50_; R:B_50:50➔89:11➔89:11_; R:B_50:50➔89:11➔50:50_; R:B_50:50➔50:50➔89:11_; and R:B_50:50➔50:50➔50:50_) (Figure 1). For each treatment and growth phase, plants were grown at 220 μmol m^−2^ s^−1^ PAR, provided by the same GreenPower Dynamic LED modules (GPL PM 168 DRBWFR L120 G3.0 C4 N4; Signify). These treatments occurred in eight individual compartments in the growth chamber, which corresponded to the eight final treatments at the end of growth phase 3.

### 2.2 Morphological Measurements

At transplant (0 DAT), 40 seedlings per cultivar were harvested as a pooled sample. At the end of each growth phase (9, 18, 27 DAT), six plants per cultivar, per treatment, per replicate experiment were imaged, harvested, and morphological measurements were performed. Photos of plants were captured with a digital camera (Nikon D7800) to visualize plant growth and pigmentation at harvest. Photos were captured with the same lens, zoom, shutter, and ISO settings in the same lighting in a windowless room. After imaging, lettuce plants were cut below the cotyledons and weighed with an analytical scale (DK‐6200‐C‐M and NL‐220‐C‐M, AllScales Europe) to measure total fresh weight. Then, leaf number was counted (leaves longer than 1 cm, excluding cotyledons) and leaf area (including cotyledons) was measured with a leaf area meter (Li‐Cor LI‐3100C, Li‐Cor Biosciences). Seedling samples (0 DAT) were weighed, measured for leaf area, and dried as a pooled sample of 40 seedlings. After leaf area was measured, all samples were immediately flash‐frozen in liquid nitrogen and stored at −80°C for at least 3 days. Harvesting occurred after 10:00 (6 h after the start of the photoperiod) until approximately 13:30. Samples were then freeze‐dried (Edwards Modulyo II, Edwards High Vacuum Int.) until completely dry, then weighed for total dry weight.

### 2.3 Sample Preparation

Individual freeze‐dried samples were stored in closed containers with desiccant to avoid re‐hydration of samples. Samples were ground to a powder using a ball mill (MM 400, Retsch). Using six freeze‐dried samples per cultivar, per treatment, per replicate experiment, 20 mg of tissue per sample was used for a pooled sample for each treatment of each replicate experiment, which was stored with desiccant until analysis. For seedlings and phase 1, all available tissue was used per treatment to create pooled samples to ensure sufficient amounts for metabolite analysis.

### 2.4 Spectrophotometric Quantification of Metabolites

#### 2.4.1 Chlorophyll, Carotenoid, and Flavonoid Determination

Chlorophyll *a*, chlorophyll *b*, total carotenoid, and total flavonoid concentrations were determined according to López‐Hidalgo et al. (2021), using ethanolic extractions of ~5 mg of the pooled freeze‐dried samples, which were weighed with a high‐accuracy balance (AT21, Mettler‐Toledo). Extraction absorbances were quantified in a 96‐well microplate (Cellstar sterile F‐bottom, Greiner Bio‐One B.V.) with a SpectraMax iD3 microplate reader (Molecular Devices). Chlorophyll *a*, chlorophyll *b*, and total carotenoid concentrations were calculated with the measured relative absorbance values at 470, 649, and 664 nm, using equations from Lichtenthaler and Buschmann (2001):

$$\mathrm{Chl}\;a=\left(13.36\cdot A_{664}\right)-\left(5.19\cdot A_{649}\right)$$

$$\mathrm{Chl}\;b=\left(27.43\cdot A_{649}\right)-\left(8.12\cdot A_{664}\right)$$

$$\mathrm{Car}=\frac{\left(1000\cdot A_{470}\right)-\left(2.13\cdot \mathrm{Chl}\;a\right)-\left(97.63\cdot \mathrm{Chl}\;b\right)}{209}$$

where *Chl a* is chlorophyll *a* (μg mL^−1^), *Chl b* is chlorophyll *b* (μg mL^−1^), *Car* is carotenoids (μg mL^−1^), and *A*_X_ is the absorbance of the sample at wavelength X.

Total flavonoids were measured at 415 nm and compared to a quercetin standard curve (from 0 to 1 mg mL^−1^); therefore, flavonoid concentration is expressed in mg quercetin equivalents per g dry weight.

#### 2.4.2 Anthocyanin Determination

Anthocyanins were extracted from ~5 mg of pooled freeze‐dried sample using acidified methanol (methanol with 1% HCl) based on Neff and Chory (1998), with chloroform separation, as in Van Brenk et al. (2025). Total relative anthocyanin concentration was calculated as relative units of (A_530_–A_657_) per gram dry weight.

### 2.5 Metabolite Analysis Using High Performance Liquid Chromatography

#### 2.5.1 Carbohydrate Determination

Samples were analyzed for their glucose, fructose, sucrose, and starch concentrations, as in van Geest et al. (2016), with modifications. Briefly, ~15 mg of pooled freeze‐dried sample was used for most samples, except ~10 mg was used for germination and phase 1 samples, due to low tissue availability. Samples were put in 12 mL centrifuge tubes with 5 mL 80% ethanol, vortexed, and heated in a shaking water bath (80°C, 20 min). Tubes were vortexed 5 s, then centrifuged (4°C, 5 min, 8500 rcf). 1 mL of supernatant was transferred to an Eppendorf tube and dried in a SpeedVac (50°C, Total time: 2 h, Heat time: 1.45 h, Pressure: 5.1 Torr; Savant SPD2010 SpeedVac Concentrator, Thermo Fisher Scientific). The remaining supernatant was stored at −20°C for starch determination. To the dried samples in Eppendorf tubes, 1 mL of Milli‐Q water was added. Tubes were vortexed 5 s, then placed in an ultrasonic water bath (22°C, 10 min). Samples were then vortexed, centrifuged (4°C, 10 min, 21,300 rcf), then diluted 10‐fold, and 1 mL was transferred to an HPLC vial for carbohydrate determination.

The remaining supernatant from carbohydrate determination (stored at −20°C) was used for starch determination. Tubes were centrifuged (4°C, 5 min, 8500 rcf); then the supernatant was discarded. To the pellet, 3 mL of 80% ethanol was added; tubes were vortexed for 5 s, centrifuged (5 min, 4°C, 8500 rcf); then the supernatant was discarded. This was repeated two more times. Samples were dried in a SpeedVac (50°C, Total time: 25 min, Heat time: 15 min, Pressure: 5.1 Torr). After drying, 2 mL of 1 mg mL^−1^ α‐amylase (in Milli‐Q water) was added to each sample and two extra tubes (blanks). Tubes were vortexed for 20 s; then put in a shaking water bath (90°C, 30 min, tubes slightly closed). Then, 1 mL amyloglucosidase (0.5 mg mL^−1^ in 50 mM citrate buffer, pH = 4.6) was added to all tubes, which were placed in a shaking water bath (60°C, 10 min). Then, tubes were centrifuged (4°C, 5 min, 8500 rcf). 1 mL of supernatant was transferred to a new Eppendorf tube, centrifuged (4°C, 10 min, 21,300 rcf); then diluted 10‐fold, and 1 mL was transferred to an HPLC vial for starch determination.

A Dionex ICS5000 HPLC was used for soluble sugars and starch analysis, using Chromeleon 7.2.10 software (equipment and software: Thermo Fisher Scientific). Two calibration sample injections with known glucose, fructose, and sucrose concentrations were used (17.60, 18.15, and 17.80 μg mL^−1^, respectively); the second injection was used to calibrate for sample quantification. 10 μL of the samples were injected into the HPLC at 25°C for determination. The method of detection was pulsed amperometric detection; the protocol runtime was 30 min, and the column was a Dionex CarboPac PA1, 250 × 2 mm with a guard column (Thermo Fisher Scientific), which separated glucose, fructose, and sucrose. In the hydrolyzed starch samples, glucose represented starch concentrations. The eluents used were 100 mM NaOH (for soluble sugars analysis) and 150 mM NaOH +50 mM NaOAc (for starch analysis), both followed by a wash step. For starch determination, a blank was added after the second calibration injection. Results in μg mL^−1^ were converted to concentrations (μg mg^−1^ dry weight) according to sample weights.

#### 2.5.2 Phenylpropanoid Determination

Samples were analyzed for their caffeic acid, chlorogenic acid, cyanidin, delphinidin, pelargonidin, and quercetin content. These metabolites were selected as compounds of interest, as they are all important phenylpropanoids, produced downstream of phenylalanine in the phenylpropanoid pathway (Dixon and Paiva 1995; Vogt 2009). These specific phenylpropanoids are responsible for the production of lignin (caffeic and chlorogenic acid), anthocyanins (cyanidin, delphinidin, pelargonidin), and flavonols/flavonoids (quercetin). Briefly, ~5 mg of pooled freeze‐dried sample was put in a 2 mL Eppendorf tube, then mixed with 500 μL 80% methanol with 1% HCl. These tubes were left for extraction in a shaker in a dark, cold room (4°C) overnight. The next day, samples were sonicated in a sonic water bath (22°C, 5 min) in the dark, then centrifuged to pellet debris (22°C, 10 min, 21,300 rcf). 100 μL supernatant was pipetted to a new Eppendorf, combined with 100 μL 2 N HCl, and incubated in a heat block (95°C, 60 min). After incubation, tubes were centrifuged (22°C, 10 min, 21,300 rcf), and 100 μL was pipetted to an amber HPLC ampule for UHPLC analysis.

An UltiMate 3000 UHPLC with a connected autosampler and Diode Array Detector was used for analysis, using Chromeleon 7.2.10 software (equipment and software: Thermo Fisher Scientific). Individual HPLC vials with standards of caffeic acid, chlorogenic acid, cyanidin, delphinidin, pelargonidin, and quercetin (Extrasynthese) were used after they received the same acidification as the samples (10 μg mL^−1^ final concentration, stocks stored at 50 μg mL^−1^ in absolute methanol). 10 μL of standard or sample was injected per vial. Phenylpropanoids were separated with a HyPURITY C18 HPLC column (150 × 3 mm, 3 μm particle size; Thermo Fisher Scientific) at a column temperature of 25°C and sampler temperature of 10°C. Using acetonitrile and 0.1% formic acid as the solvents, the flow gradient of acetonitrile was performed as follows: 0–15 min, 5%–35%; 15–20 min, 35%; 20–30 min, 35%–40%; 30–35 min, 40%; 35–40 min, 40%–50%; 40–52 min, 50%–70% (all linear gradients with flow rate 0.45 mL min^−1^). Then, the column was cleared for the next injection by decreasing acetonitrile to 5% between 52 and 60 min, then kept at 5% from 60 to 70 min (both linear, 0.45 mL min^−1^).

Peak retention times and spectra of extracted samples were compared to the spectral characteristics of standards to determine phenylpropanoid content. The Diode Array Detector was used to monitor three wavelengths for the assorted phenylpropanoids: 310 nm for caffeic acid and chlorogenic acid; 360 nm for quercetin; and 525 nm for cyanidin, delphinidin, and pelargonidin.

### 2.6 Cell Wall Purification

Preceding lignin analysis, cell walls were purified from the pooled freeze‐dried samples. Briefly, ~15 mg of pooled sample was used to obtain at least 5 mg of cell wall, as our tests yielded ~40% of the original sample weight after processing. Cell wall preparation was performed with sequential washes using 1 mL of different solutions, as in Corneillie et al. (2019). Washes were incubated in a shaking heat block (Eppendorf ThermoMixer Compact) for 30 min at different temperatures, in the following order: water (98°C), absolute ethanol (76°C), chloroform (59°C), and acetone (54°C). After each incubation, samples were centrifuged (5 min, 13,300 rpm); then pellets were isolated, removing supernatants with careful pipetting. After the final supernatant removal, samples were placed in a fume hood overnight for acetone evaporation. Pellets were further dried using a RapidVap Vacuum Dry Evaporation System (Labconco) connected to a −50°C CentriVap Cold Trap (Labconco). Samples were weighed after extraction, and cell wall content was calculated as a percent of the initial sample weight.

### 2.7 Lignin Analysis

#### 2.7.1 Thioacidolysis

Using the purified cell walls of the pooled samples, lignin composition was determined in accordance with a rapid and high‐throughput procedure from (Chen, Zhuo, et al. 2021). Approximately 5 mg from the purified cell walls was transferred to 2 mL micro‐reaction glass vials with secure caps. 1 mL of thioacidolysis reagent (2.5% boron trifluoride diethyl etherate, 10% ethanethiol, 87.5% dioxane) was added to all samples. Vials were placed in a preheated (100°C) 80‐position MULTIVAP Nitrogen Evaporator (Organomation). After 4 h, with hourly manual shaking of the vials, 400 μL of thioacidolyzed samples were moved to 2 mL Eppendorf tubes containing 190 μL of saturated NaHCO_3_ to stop the reaction. This was repeated in another tube to prepare a duplicate backup of the thioacidolyzed sample for derivatization. All tubes were opened and placed in the preheated (55°C) 80‐position MULTIVAP Nitrogen Evaporator to dry completely overnight using nitrogen gas. The next morning, samples were transferred to a desiccator for 1 week to allow for complete dehydration. After thioacidolysis and drying, derivatization and gas chromatography–mass spectrometry (GC–MS) analysis were performed.

#### 2.7.2 Derivatization and GC–MS

A 200 μL of derivatization reagent [1:1 pyridine and BSTFA + 1% TMCS Silylation Reagent (v/v)] was added to each dried thioacidolyzed sample, mixed in a shaking heat block (55°C, 2 h), then left to cool. After cooling, samples were centrifuged (22°C, 5 min, 21,300 rcf) and 80 μL of derivatized sample was transferred to GC crimp vials with glass inserts. Vials were placed in the GC–MS system (Trace GC Ultra with Trace DSQ, Thermo Fisher Scientific) for quantification of individual lignin monomers using an HP‐5MS column (30 m × 0.25 mm × 0.25 μm; Agilent Technologies). GC–MS settings were the same as (Chen, Zhuo, et al. 2021), except with an injection volume of 3 μL instead of 2 μL. Total lignin content was calculated as the sum of thioacidolysis monomer yields.

### 2.8 Energy‐Use Efficiency and Phytochemical Production Efficiency Calculations

Energy‐use efficiency (g FW·kWh^−1^) was calculated per treatment using plant fresh weight, planting density (73 plants m^−2^), duration of R:B treatments (27 days), and total radiation received within 400–700 nm. Phytochemical production efficiencies of anthocyanins and flavonoids were calculated using concentrations per gram fresh weight (e.g., mg·g^−1^ FW·kWh^−1^), dry weight (mg·g^−1^ DW·kWh^−1^), and content per plant (e.g., mg·plant^−1^ kWh^−1^). Calculations were performed using photon efficacy values of typical LED packages (*R* = 4.1 μmol J^−1^ and *B* = 2.8 μmol J^−1^) from Kusuma et al. (2022).

### 2.9 Statistical Design and Analysis

The experiment was conducted four times, resulting in a completely randomized design with four blocks (*n* = 4). In each experiment, light treatments were randomized over the growth compartments. Data analysis was performed with RStudio (PBC, Boston) and Genstat (21st edition, VSN International LTD). For every variate, normality was checked with the Shapiro–Wilk test and homogeneity with Bartlett's test. If the data passed both tests, a one‐way analysis of variance (ANOVA) with blocks was conducted for each measured parameter using the treatment as the factor within each phase. For every measurement, a protected Fisher's LSD test was then performed to assess the significance of differences between group means (*α* = 0.05).

## 3 Results

### 3.1 Increased Exposure to Low Red:Blue Reduces Plant Size and Weight, but Not Dry Matter Content

**FIGURE 2:**
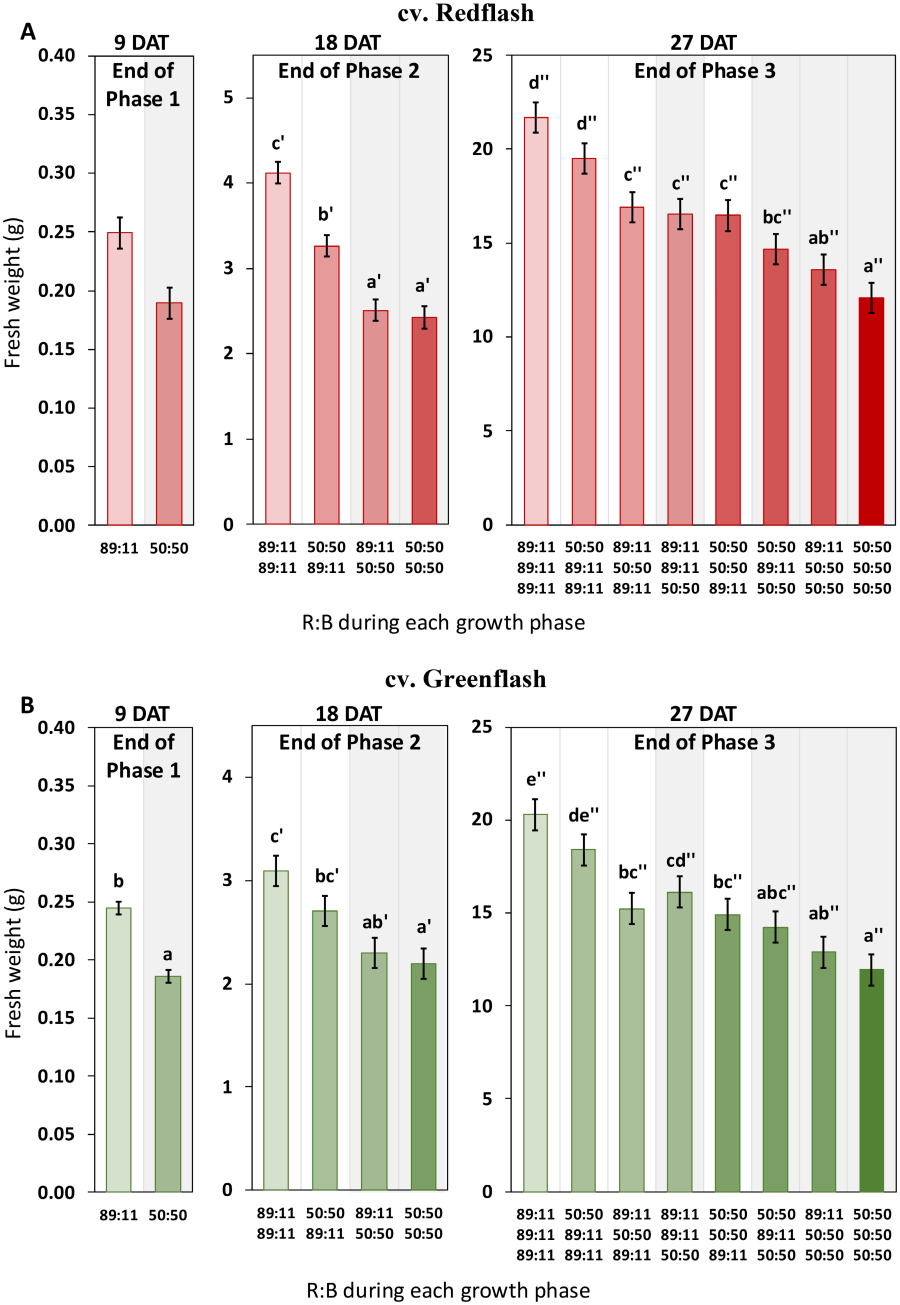
Effects on fresh weight from 9‐day periods of high or low red:blue ratio during three different phases of cultivation for two lettuce cultivars. Fresh weight (g) of lettuce cv. Redflash (A–C) and cv. Greenflash (D–F) grown for up to 27 days after transplant. Two different R:B ratios (R:B_89:11_ and R:B_50:50_) were applied during each of three 9‐day phases of growth (Phase 1, Phase 2, and Phase 3). Datapoints represent means with standard error means of four growth cycles (*n* = 4), each consisting of six replicate plants. A one‐way ANOVA was performed within each phase. Different letters indicate significantly different values for each treatment within a phase, according to a protected Fisher LSD test (*a* = 0.05), for Phase 1 (no apostrophe), Phase 2 (′), and Phase 3 (″). The color and order of the data bars correspond with the number of phases that low R:B was applied, with a gray background indicating a final phase of low R:B.

**FIGURE 3:**
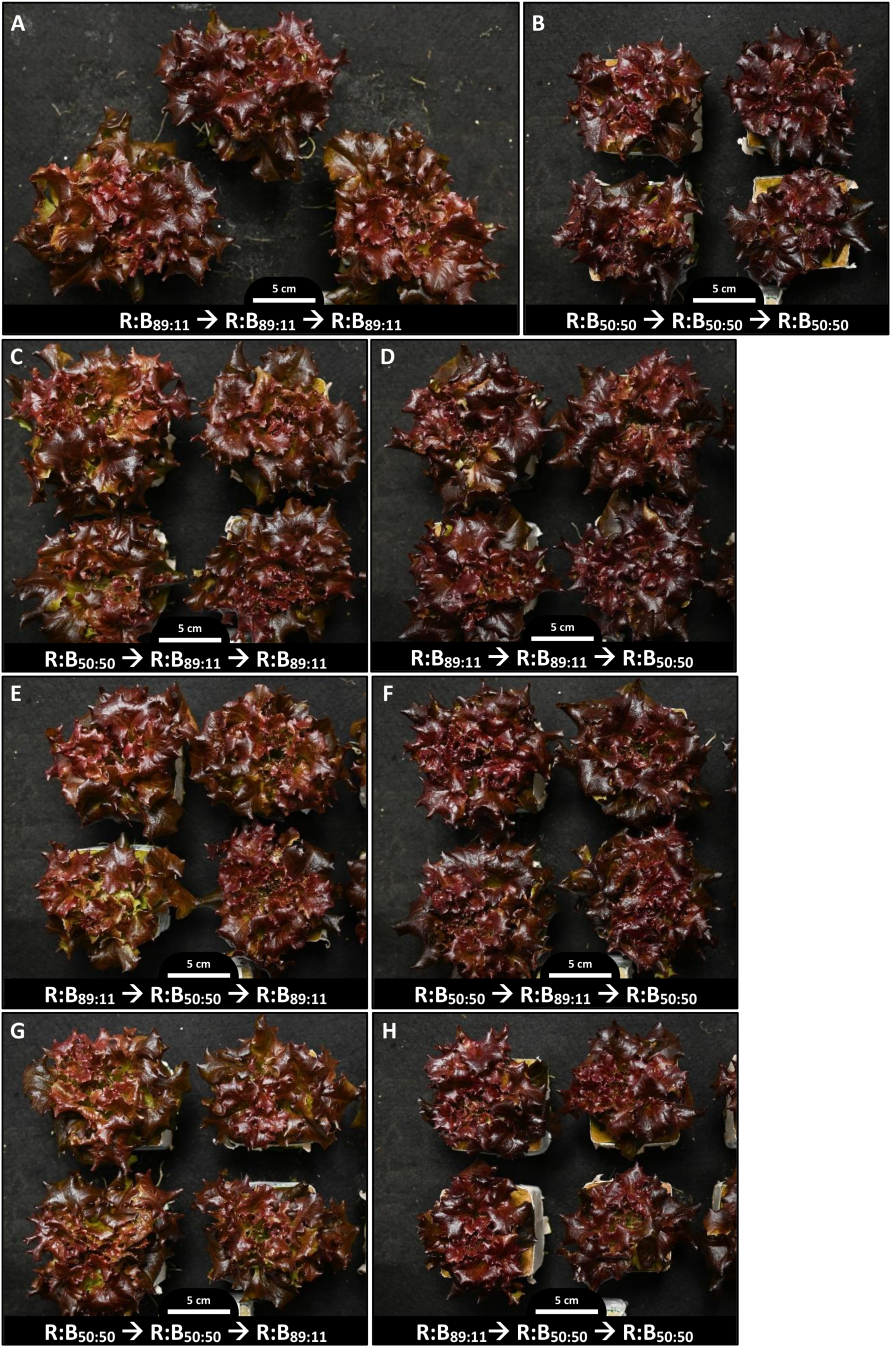
Photos of cv. Redflash lettuce grown under high or low red:blue ratio for three phases. Representative visual appearance (overhead photos) of cv. Redflash at 27 days after transplant (DAT), displaying the effects of red:blue photon ratios applied during phase 1 (0–9 DAT), phase 2 (9–18 DAT) and phase 3 (18–27 DAT). Two different R:B ratios (R:B_89:11_ and R:B_50:50_) were applied during these 9‐day phases of growth; denoted at the bottom of each photograph is the red:blue ratio used in each phase of plant growth (Phase 1 ➔ Phase 2 ➔ Phase 3).

**FIGURE 4:**
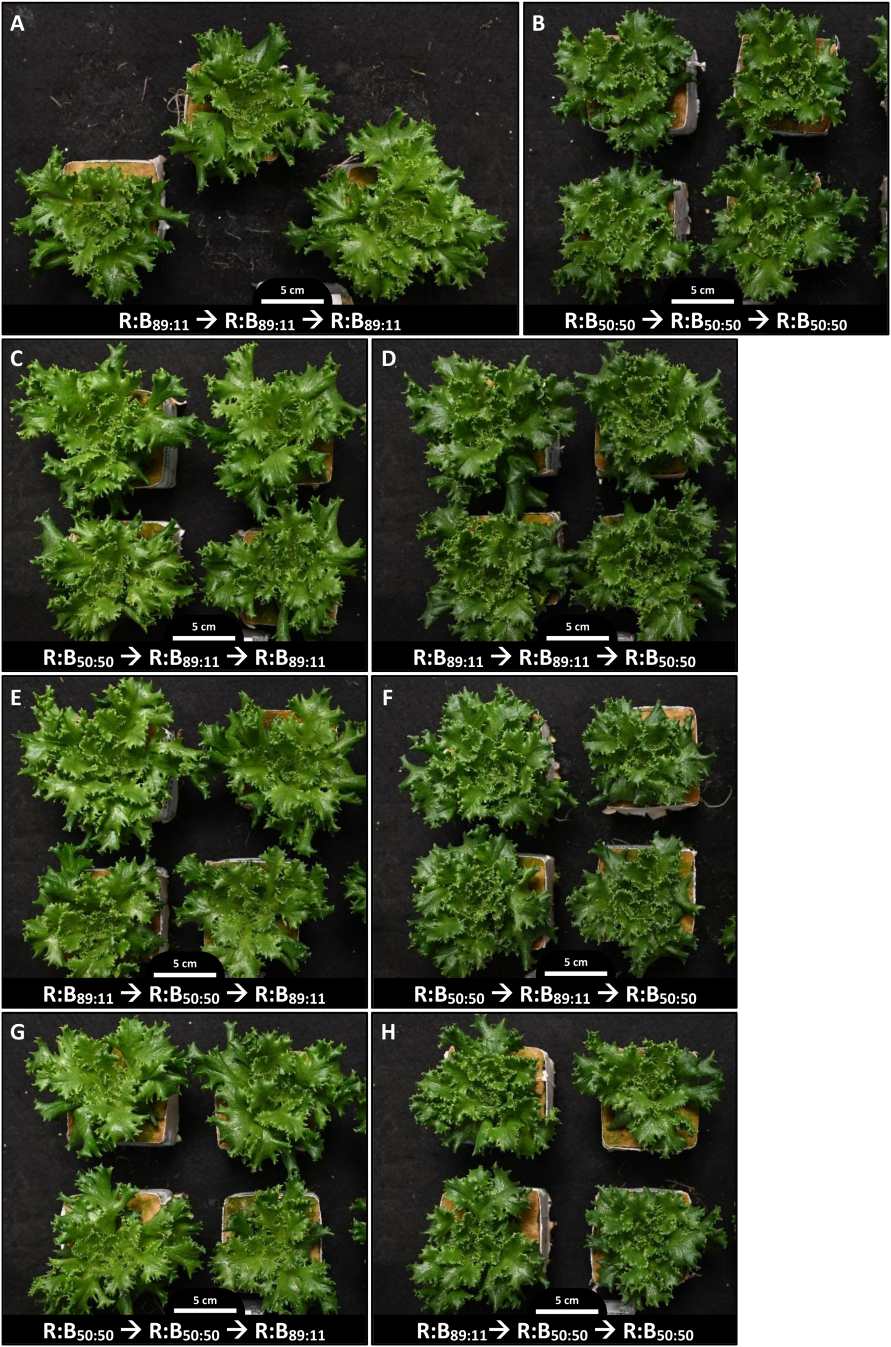
Photos of cv. Greenflash lettuce grown under high or low red:blue ratio for three phases. Representative visual appearance (overhead photos) of cv. Greenflash at 27 days after transplant (DAT), displaying the effects of red:blue photon ratios applied during phase 1 (0–9 DAT), phase 2 (9–18 DAT) and phase 3 (18–27 DAT). Two different R:B ratios (R:B_89:11_ and R:B_50:50_) were applied during these 9‐day phases of growth; denoted at the bottom of each photograph is the red:blue ratio used in each phase of plant growth (Phase 1 ➔ Phase 2 ➔ Phase 3).

After one growth phase (9 days after transplant; DAT), plants grown under high R:B already showed greater fresh weight, dry weight, and leaf area compared to those grown with low R:B (Figures 2, S1A,B and S2A,B; Table S1). This was true for both cultivars, although Redflash fresh weight at 9 DAT was not significantly different (*p =* 0.051). At the end of phase 2 (18 DAT), growth was highest for plants grown only under high R:B, followed by plants that received high R:B after a phase of low R:B (Figures 2, S1C–F and S2C–F; Table S1). Plants that received low R:B in phase 2 (R:B_89:11_ ➔ R:B_50:50_ and R:B_50:50_ ➔ R:B_50:50_) had the lowest growth parameters at 18 DAT (Figures 2, S1C–F, and S2C–F; Table S1). Continuing this trend, at the end of phase 3 (27 DAT), plants that received constant high R:B (R:B_89:11_) had the greatest fresh weight, dry weight, and leaf area (Figures 2, 3, 4; Table S1). Conversely, plants grown under constant low R:B (R:B_50:50_) had the lowest values for these growth parameters (Figures 2, 3, 4; Table S1). Dynamic treatments resulted in growth that aligned with increased total exposure to low R:B (Figures 2, 3, 4; Table S1). That is, plants grown with one phase of low R:B (e.g., R:B_50:50_ in phase 1, but R:B_89:11_ in phases 2 and 3) had higher fresh weight, dry weight, and leaf area than those grown under two phases of low R:B (e.g., R:B_89:11_ in phase 1, R:B_50:50_ in phases 2 and 3). Further, plants grown under low R:B in any of the final two phases had reduced growth compared to those that received low R:B in the first phase.

In Redflash, dry matter content was not significantly affected by R:B treatments but decreased with growth phases (Table S1). Greenflash dry matter content was marginally increased by low R:B in the last phase but had no significant differences due to R:B in other phases (Table S1). Redflash specific leaf area did not significantly change with R:B treatment, but Greenflash plants had slightly lower specific leaf area in low R:B than under high R:B in phase 3 (Table S1). Redflash had fewer leaves with constant low R:B compared to high R:B, but the number of Greenflash leaves was not significantly affected by R:B treatments (Table S1).

### 3.2 Low R:B Exposure Before Harvest Causes Darker Leaf Pigmentation and a Higher Concentration of Pigment‐Associated Compounds

Any plant grown under low R:B for the last phase of growth had darker pigmentation (darker purple red in Redflash and darker green in Greenflash) compared to plants grown under high R:B for the last phase (Figures 3, 4, S1 and S2). These effects on lettuce pigmentation can be a product of different pigmented metabolites, including chlorophyll, flavonoids, and anthocyanins.

#### 3.2.1 Chlorophyll Increases With Low Red:Blue

**FIGURE 5:**
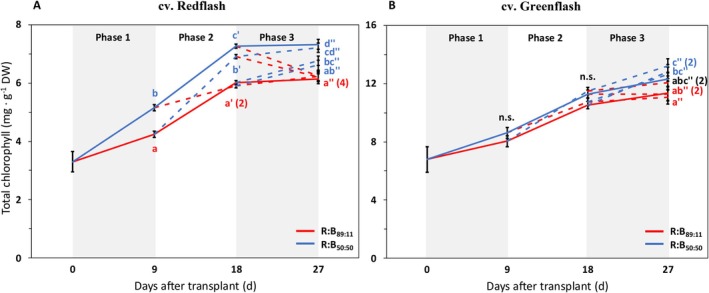
Effects on total chlorophyll from 9‐day periods of high or low red:blue ratio during three different phases of cultivation for two lettuce cultivars. Total chlorophyll (mg·g^−1^) in cv. Redflash (A) and cv. Greenflash (B) grown for a total of 27 days after transplant, with three 9‐day phases of receiving either R:B_89:11_ (red line) or R:B_50:50_ (blue line). Solid lines represent constant treatments of R:B_89:11_ or R:B_50:50_ for the whole growth cycle, dashed lines represent treatments that transition between the two R:B ratios. A one‐way ANOVA was performed within each phase. Datapoints represent means with standard error means of four growth cycles (*n* = 4), each consisting of six replicate plants. Different letters indicate significantly different values for each treatment within a phase, according to a protected Fisher LSD test (*a* = 0.05), for Phase 1 (no apostrophe), Phase 2 (′), and Phase 3 (″).

Redflash had increased chlorophyll with low R:B for each phase (Figure 5A). Greenflash also had increased chlorophyll under low R:B, but this was only significantly different in plants after the last phase (Figure 5B). All plants that received low R:B in the last growth phase had higher chlorophyll than those grown under high R:B (Figure 5). Total chlorophyll content of both cultivars increased over time, and Redflash had lower total chlorophyll content than Greenflash (Figure 5). These patterns align with the individual chlorophylls, *a* and *b* (Table S2); additionally, the chlorophyll *a:b* ratio did not significantly change with R:B treatment (Table S2). R:B had no significant effect on carotenoids for each phase of growth (Table S2).

#### 3.2.2 Lower Red:Blue Increases Flavonoids

**FIGURE 6:**
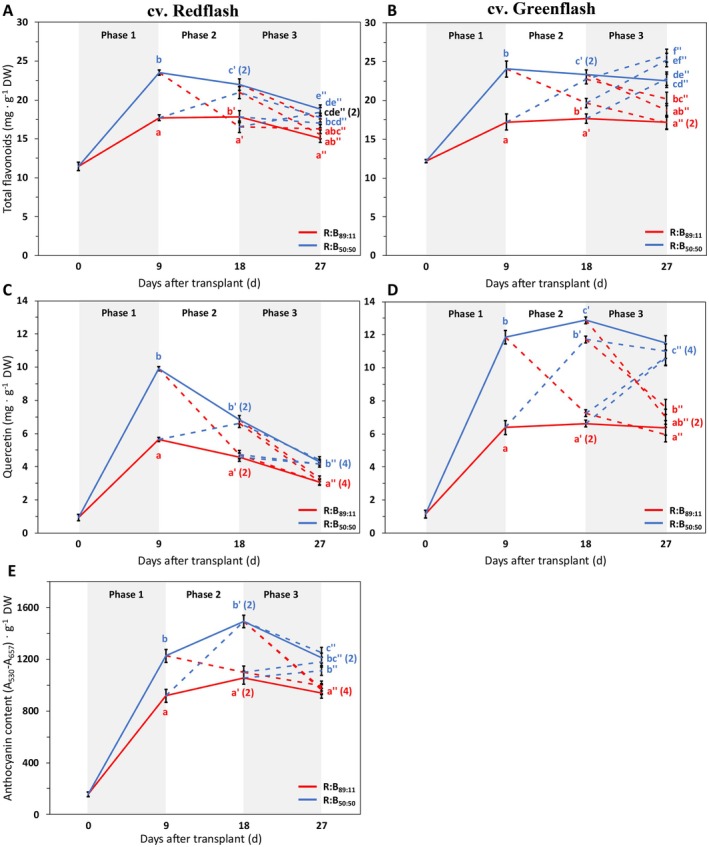
Effects on total flavonoids, quercetin, and anthocyanins from 9‐day periods of high or low red:blue ratio during three different phases of cultivation for two lettuce cultivars. Total flavonoid concentration (A, B; mg·g^−1^), quercetin concentration (C, D; mg·g^−1^), and relative anthocyanin content (E, A_530_–A_657_·g^−1^) in cv. Redflash (A, C, E) and cv. Greenflash (B, D) grown for a total of 27 days after transplant, with three 9‐day phases of receiving either R:B_89:11_ (red line) or R:B_50:50_ (blue line). Solid lines represent constant treatments of R:B_89:11_ or R:B_50:50_ for the whole growth cycle, dashed lines represent treatments that transition between the two R:B ratios. A one‐way ANOVA was performed within each phase. Datapoints represent means with standard error means of four growth cycles (*n* = 4), each consisting of six replicate plants. Different letters indicate significantly different values for each treatment within a phase (*a* = 0.05), for Phase 1 (no apostrophe), Phase 2 (′), and Phase 3 (″).

In each phase, high R:B always resulted in less total flavonoids and quercetin than low R:B (Figure 6A–D). When low R:B was applied after high R:B, quercetin and flavonoids increased, more strongly in Greenflash than Redflash (Figure 6C,D). Conversely, quercetin and flavonoids decreased whenever high R:B was applied after low R:B (Figure 6C,D). Notably, quercetin strongly increased in both cultivars during the first growth phase, where low R:B doubled the quercetin content of high R:B (Figure 6C,D). During the second and third phases, Redflash quercetin decreased with time, whereas Greenflash maintained high concentrations under low R:B or low concentrations under high R:B (Figure 6C,D). This was also seen with flavonoids, but to a lesser extent (Figure 6A,B).

#### 3.2.3 Higher Anthocyanins Are Produced at Lower Red:Blue

Anthocyanin content in Redflash (Figure 6E) showed a very similar pattern to that of quercetin in Greenflash (Figure 6D). These contents were higher with low R:B compared with exposure to high R:B for every phase (Figure 6D,E). Again, there was also a strong increase of anthocyanins in the first phase of growth, which was higher with low R:B. Anthocyanins had the highest concentrations after the second growth phase. This was seen for total anthocyanins analyzed with spectrophotometry (Figure 6E) and was also seen for the individual anthocyanin subcomponents, cyanidin and pelargonidin, analyzed with HPLC (Figure S3). Cyanidin was found to be the greatest contributor to total anthocyanins, with a much higher concentration than pelargonidin. Delphinidin, another precursor of other anthocyanins, was not detected in our analysis.

### 3.3 Carbohydrate Content Decreases by Low Red:Blue

**FIGURE 7:**
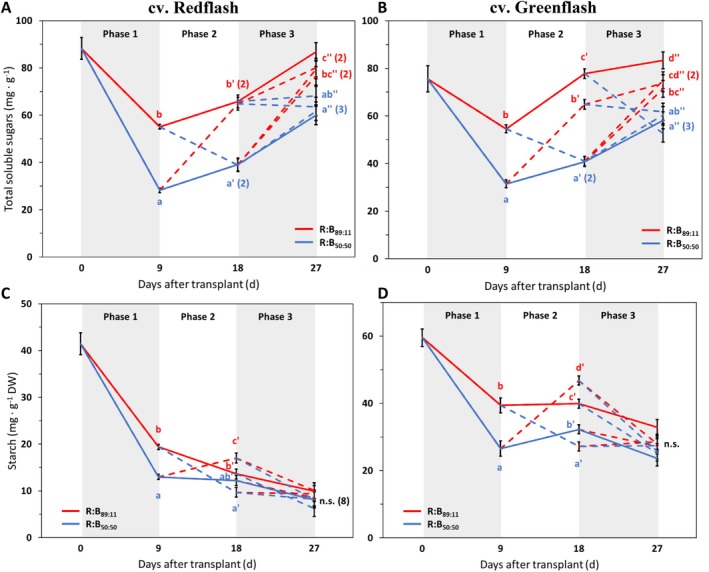
Effects on total soluble sugars and starch from 9‐day periods of high or low red:blue ratio during three different phases of cultivation for two lettuce cultivars. Total soluble sugars (A, B; mg·g^−1^) and starch (C, D) in cv. Redflash (A) and cv. Greenflash (B) grown for a total of 27 days after transplant, with three 9‐day phases of receiving either R:B_89:11_ (red line) or R:B_50:50_ (blue line). Solid lines represent constant treatments of R:B_89:11_ or R:B_50:50_ for the whole growth cycle, dashed lines represent treatments that transition between the two R:B ratios. A one‐way ANOVA was performed within each phase. Datapoints represent means with standard error means of four growth cycles (*n* = 4), each consisting of six replicate plants. Different letters indicate significantly different values for each treatment within a phase, according to a protected Fisher LSD test (*a* = 0.05), for Phase 1 (no apostrophe), Phase 2 (′), and Phase 3 (″).

Carbohydrates had the opposite pattern of flavonoids in response to R:B (Figure 7A,B). For each growth phase, high R:B application produced higher carbohydrate concentrations than low R:B in both cultivars (Figure 7A,B). Constant high R:B exposure resulted in the highest total soluble sugar concentration, and constant low R:B corresponded with the lowest concentration. Strikingly, Redflash soluble sugars increased with high R:B for each phase, without being affected by prior periods (Figure 7A,B); the converse was also true: low R:B caused the same (lower than high R:B) sugar content for each phase, regardless of prior growth spectra (Figure 7A,B). These patterns of total soluble sugar concentration were due to the sum of its parts, as they were seen for each of sucrose (Figure S4A,B), glucose (Figure S4C,D), and fructose (Figure S4E,F). Low R:B reduced starch only during phase 1 and phase 2; R:B treatments during phase 3 showed no significant differences in starch for either cultivar (Figure 7C,D).

### 3.4 Cell Wall Content and Lignin Do Not Appear to Be Affected by Red:Blue Light Treatments

No consistent treatment effects on cell wall content were discerned for either cultivar (Table S3). Importantly, both cultivars also had no significant change in cell‐wall‐associated lignin components for any R:B treatment or phase for the analyzed monomers (guaiacyl—G, or syringyl—S monolignols). Furthermore, the S/G ratio was also not significantly affected by R:B for any growth phase for both cultivars. Notably, no p‐hydroxyphenyl (H) lignin monomers were detected in our analysis (below detection limit) in line with previous findings that excluded the role of this monomer for lignin composition in angiosperm dicots such as lettuce (Vanholme et al. 2010). Finally, there were no treatment effects from R:B on caffeic and chlorogenic acid concentrations for both cultivars. These were unexpected results; each of these analyzed cell wall‐associated compounds was unaffected by the tested R:B ratios, whereas flavonoids and carbohydrate contents were strongly impacted by R:B ratio.

### 3.5 Energy‐Use Efficiency and Phytochemical Production Efficiency Contrast Each Other

In both cultivars, the energy‐use efficiency was highest under constant high R:B and lowest under constant low R:B (Table 1). With increased high R:B exposure, the energy‐use efficiency of dynamic treatments increased (Table 1). That is, plants grown under two high R:B phases and one low R:B phase had higher energy‐use efficiency (40–50 g FW·kWh^−1^) than plants grown under one high R:B phase and two low R:B phases (32–40 g FW·kWh^−1^). Phytochemical production efficiency was also calculated using flavonoid and anthocyanin content at harvest (Tables 1 and S4). Flavonoids in Greenflash had higher production efficiency under treatments with a final phase of low R:B, rather than with a final phase of high R:B, including constant high R:B (Tables 1 and S4). This also appeared to be the trend for Redflash flavonoid concentrations, but this was not found to be significant based on treatments. However, Redflash did produce anthocyanins with more efficiency in each treatment with a final phase of low R:B (Tables 1 and S4).

**TABLE 1:** Energy‐use efficiency and phytochemical production efficiency from 9‐day periods of high or low red:blue ratio during three different phases of cultivation for two lettuce cultivars.

R:B* during days			Energy‐use efficiency $\left(\frac{g\;\mathrm{FW}}{\mathrm{kWh}}\right)$	Phytochemical production efficiency	
0–9 (Phase 1)	10–18 (Phase 2)	19–27 (Phase 3)		Flavonoids mggFWkWh	Anthocyanins** AgFWkWh
*cv. Redflash at 27 DAT*					
89:11	89:11	89:11	58.5^f^	2.57	160.4^a^
50:50	89:11	89:11	50.4^e^	2.73	166.7^ab^
89:11	50:50	89:11	43.7^d^	2.60	162.5^a^
50:50	50:50	89:11	40.7^cd^	2.77	152.9^a^
89:11	89:11	50:50	42.8^d^	2.83	185.8^bc^
50:50	89:11	50:50	36.2^bc^	2.99	190.8^c^
89:11	50:50	50:50	33.6^ab^	2.86	201.9^c^
50:50	50:50	50:50	28.4^a^	2.90	186.4^bc^
**SEM**			2.08	0.10	6.98
***P***_***Treatment***_			**< 0.001**	0.118	**< 0.001**
*cv. Greenflash at 27 DAT*					
89:11	89:11	89:11	54.8^f^	3.10^a^	N/A
50:50	89:11	89:11	47.6^e^	3.06^a^	N/A
89:11	50:50	89:11	39.4^cd^	3.60^bc^	N/A
50:50	50:50	89:11	36.9^bcd^	3.16^ab^	N/A
89:11	89:11	50:50	41.7^de^	4.20^de^	N/A
50:50	89:11	50:50	35.1^bc^	4.52^e^	N/A
89:11	50:50	50:50	31.8^ab^	4.50^e^	N/A
50:50	50:50	50:50	28.1^a^	3.74^cd^	N/A
**SEM**			2.12	0.16	N/A
***P***_***Treatment***_			**< 0.001**	**< 0.001**	N/A

*Note:* Data are means of four growth cycles (*n* = 4), each consisting of six replicate plants. A one‐way ANOVA was performed. Different letters indicate significantly different values for each treatment within a phase, according to a protected Fisher LSD test (*a* = 0.05). Bolded numbers indicate the probability of a significant effect from R:B treatments.

Abbreviations: DAT, days after transplant; FW, fresh weight; kWh, kilowatt‐hour; N/A, not applicable; *P*_*Treatment*_, probability of an effect from R:B treatments; R:B, red:blue light ratio; SEM, standard error of means.

* The indicated R:B was applied during three phases that lasted 9 days.

** For anthocyanins, relative absorbance (A) was calculated as A_530_–A_657_.

## 4 Discussion

### 4.1 Low R:B Negatively Impacts Growth, With a Greater Impact When Applied Later in Cultivation

The R:B spectra that plants received significantly impacted growth and morphology (Figures 2, 3, 4, S1 and S2; Table S1). Our previous findings indicated that high R:B (R:B_89:11_) over the entirety of cultivation enhanced growth and biomass accumulation, while low R:B (R:B_50:50_) increased pigment production and potentially nutritional quality (Van Brenk et al. 2024, 2025). Several studies also report higher growth under high R:B (Azad et al. 2020; Miao et al. 2019; Samuolienė et al. 2021), and Son et al. (2017) found that decreasing R:B over time reduced growth compared to constant high R:B. However, their treatments did not differentiate total B exposure from early or late exposure to low R:B. The unique dynamic spectra applications in the present experiment showed that lettuce weight, leaf area, and size decreased the longer that lettuce was grown with low R:B (Figures 2, 3, 4, S1 and S2; Table S1). Thus, we confirm that low R:B negatively impacts lettuce growth the longer that low R:B is applied, with greater impact on final plant weight if low R:B is applied in later stages of cultivation. Jin et al. (2023) found lettuce yield increased with increased end‐of‐cultivation light intensity, attributed to more light interception and absorption from larger leaves in later production, allowing for more growth. The R:B spectra in the last phases of production similarly influence growth, as plants grown under high R:B at the end of production have larger leaves and thus, higher light interception. Photosynthetic assimilation rate (both overall and per unit leaf area) also increases as R:B increases (Van Brenk et al. 2025; Wang et al. 2016). With these combined factors, R:B application impacts growth more when manipulated nearer to the end of production.

### 4.2 Lettuce Pigmentation at Harvest Is Determined by the Light Spectrum Shortly Before Harvest

Lettuce coloration at harvest is an important factor in consumer preference; here, plant pigments at harvest were largely dependent on the most recent application of R:B, independent of the preceding phases' R:B spectra. For each growth phase, plants had lighter pigment under high R:B and darker pigment from low R:B (Figures 3, 4, S1, and S2), aligning with increased plant pigments under low R:B, namely chlorophyll (Figure 5), flavonoids (Figure 6A–D), and anthocyanins specifically in red lettuce (Figure 6E). As plant pigments, the presence of each of these metabolites affects plant color (Khoo et al. 2017; Kong and Nemali 2021; Naznin et al. 2019); their contents and responses to R:B are discussed in the following sections.

### 4.3 The Final Induction of Chlorophyll and Flavonoids With Low Red:Blue Is Most Impactful

Chlorophyll (Figure 5), flavonoid (Figure 6A,B), and quercetin concentrations (Figure 6C,D) increased under low R:B and decreased under high R:B, agreeing with previous work showing increased chlorophyll and flavonoid concentrations under low R:B (Hogewoning et al. 2010; Son et al. 2017; Son and Oh 2013; Van Brenk et al. 2024). Our results with dynamic treatments are supported by Son et al. (2017), who found that transitioning growth spectra from monochromatic R to monochromatic B induced chlorophyll, phenolic compounds, and flavonoid concentrations. In our study, we found that whenever plants received low R:B after a high R:B phase, chlorophyll and flavonoid concentrations increased (or decreased if high R:B was applied after low R:B). These increases of chlorophyll and flavonoids with decreasing R:B can be a result of regulation of genes associated with their biosynthesis and through photoreceptor responses. For chlorophyll, B light has been reported to increase the concentrations of chlorophyll biosynthesis intermediates and alleviate the R light‐induced reduction of chlorophyll biosynthesis (Fan et al. 2013). For flavonoids, cryptochrome receptors are activated by B light, which activates HY5 by reducing COP1 inhibition of HY5 (Ma et al. 2021; Tissot and Ulm 2020; Yu et al. 2010). HY5 then induces the expression of chalcone synthase and flavonol synthase, enzymes in the phenylpropanoid pathway that synthesize flavonoids (Ma et al. 2021; Sng et al. 2021; Tissot and Ulm 2020). While these responses to B have been well described, there has been limited analysis on dynamic R:B applications considering the responses of chlorophyll and flavonoid accumulation. Importantly, we found that plants that received low R:B in the final phase of growth had high chlorophyll and flavonoid concentrations, similar to those of static treatments (i.e., a transition to low R:B resulted in concentrations as if the plant had always received low R:B, and vice versa). Altogether, the spectra exposed to plants during the final growth phase surpass the impact of any preceding growth spectra for chlorophyll and flavonoid content.

### 4.4 Low R:B Induces Phenylpropanoid Accumulation, Leading to More Anthocyanins in Red Lettuce and More Quercetin‐Derived Flavonoids in Green Lettuce

Interestingly, the red and the green lettuce cultivars had different concentrations of quercetin‐derived flavonoids (Figure 6C,D). Green lettuce had two‐fold higher quercetin levels when grown under low R:B compared with high R:B, regardless of the age of the plant (Figure 6D). On the other hand, red lettuce quercetin was always higher under low R:B than high R:B, but (following an initial increase) quercetin content in red lettuce decreased as the plant aged (Figure 6C). This begs the question, why does the quercetin concentration decrease in red lettuce and not in green lettuce? As quercetin‐derived flavonoids can address the harmful effects caused by ROS (Pietta 2000; Williams et al. 2004), would both cultivars not benefit from quercetin presence? These questions may be answered by considering anthocyanins, the pigments that distinguish red and green cultivars.

Anthocyanins in red lettuce had strikingly similar patterns of response to R:B when compared to quercetin concentrations in green lettuce (Figures 6D,E and S3). Importantly, in transitions from high to low R:B, red lettuce anthocyanin content increased to a level equal to that of plants grown under static low R:B (Figures 6E and S3). Conversely, transitioning from low to high R:B, anthocyanins decreased to levels of static high R:B. Anthocyanins filter harmful light, in addition to their inherent ROS‐scavenging traits as flavonoid antioxidants. Thus, increased anthocyanins reduce potential ROS formation, while also scavenging the (fewer) ROS that do form. In this study, increased anthocyanin content alongside decreased quercetin content in red lettuce indicates several points: (1) anthocyanin light filtering in red lettuce outweighs the need to address ROS with quercetin‐derived metabolites; (2) green lettuce, which produces low or even no anthocyanins, relies more heavily on producing quercetin‐derived metabolites to address ROS; as a result, (3) green lettuce produces more quercetin over time (especially in low R:B) at notably higher levels compared to red lettuce. Quercetin and anthocyanin biosynthesis share common precursors (Bentley 1990; Herrmann and Weaver 1999; Vogt 2009), but the synthesis enzymes of anthocyanins (e.g., anthocyanin synthase; Wilmouth et al. 2002) may not be functional or produced in green lettuce. In summary, red and green lettuce both upregulate phenylpropanoid production in response to low R:B, but by using different flavonoids. Red lettuce and green lettuce therefore utilize different approaches and metabolic routes to culminate in the specific end products of these protective compounds.

### 4.5 Lignin, Cell Wall Content, Caffeic Acid, and Chlorogenic Acid Remain Stable Under Combinations of Red:Blue Light

Based on prior research, we also aimed to determine if the resources allocated to flavonoids under low R:B would reduce lignin production. Firstly, plant growth under low R:B is impaired while flavonoid production increases (Van Brenk et al. 2024). Secondly, flavonoids share phenylpropanoid precursors and regulatory biosynthesis elements with lignin (Dixon and Paiva 1995; Zhang et al. 2021). Thirdly, lignin is associated with cell wall structure and therefore (to some extent) growth (Liu et al. 2018; Vanholme et al. 2010). Previous studies on light quality effects on lignin were primarily under foliar shade or shade‐mimicking conditions with far‐red light (Hussain et al. 2019; Ranade et al. 2022; Wu et al. 2017), hypocotyls under monochromatic B (Wang et al. 2023), or were specific to lignin in fruit stone cells (Wang et al. 2022). These studies are necessary forays to link B and lignin, but there remain few studies on spectra and lignin in horticulture. Hence, it was valuable to determine potential trade‐offs between spectra, growth, and quality considering lignin and other phenylpropanoids.

Here, R:B spectra did not significantly affect lignin composition, lignin monomers, caffeic acid, or chlorogenic acid (Table S3). In addition to other functions (e.g., wounding response), caffeic acid and chlorogenic acid are phenylpropanoids and precursors in lignin biosynthesis (Dixon and Paiva 1995). Although it was surprising that lignin, caffeic acid, and chlorogenic acid were all insensitive to R:B variation, this indicates that plants under low R:B may therefore still produce cell walls with sufficient lignin per gram, even if phenylpropanoid pathway resources are channeled toward flavonoids. Although studies have shown B increases caffeic and chlorogenic acid, these only compared monochromatic B and R light (Shimomura et al. 2020; Son et al. 2017). Like our results, Son et al. (2017) showed that caffeic and chlorogenic acid increased with decreased R:B, but not significantly. Further, R:B had no clear effect on cell wall content (Table S3). These results indicate (within this study's R:B spectra) that phenylpropanoid production can ensure cell wall stability via consistent production of lignin and its metabolic precursors, all while increasing flavonoids. Early reactions in the phenylpropanoid pathway (preceding lignin, flavonoid, and cell wall metabolite biosynthesis) are unaffected by these R:B spectra; instead, later branches toward flavonoid production are upregulated. This suggests that the regulation of lignin and flavonoid biosynthesis may be individually controlled, or at least decoupled, under these R:B conditions, even though these phenylpropanoids share common biosynthesis precursors.

### 4.6 Carbohydrates Are Induced Under High R:B, Resulting in Increased Growth Potential

Carbohydrates are the main assimilates for energy (Apriyanto et al. 2022; Rosa et al. 2009); they are connected to phenylpropanoid synthesis via the shikimate pathway, which links central carbon metabolism to aromatic amino acid synthesis (Maeda and Dudareva 2012). Carbohydrates derived from photosynthesis include soluble sugars (e.g., glucose, fructose, and sucrose) and starch as an insoluble carbohydrate (Apriyanto et al. 2022; Smith and Stitt 2007). We found that high R:B always produced more soluble sugars than plants grown under low R:B (Figures 7A,B, S4 and S5). Increased carbohydrate pools under high R:B contribute to increased plant growth (Van Brenk et al. 2024). Reduced carbohydrates under low R:B can be from reduced photosynthesis (Van Brenk et al. 2025), or can indicate that more carbon is utilized—low carbohydrates may be a result of increased production of specialized metabolites. Regardless, plants under low R:B likely assimilate less carbon from photosynthesis (Liu and van Iersel 2021; Van Brenk et al. 2025; Wang et al. 2016), yet still produce increased concentrations of certain specialized metabolites with this reduced carbon (Naznin et al. 2019; Sng et al. 2021; Van Brenk et al. 2025; J. Wang et al. 2016). Initially, we postulated that reduced plant growth may be due to reduced lignin concentration, but this was not the case. Instead, the reduced energy from fewer carbohydrates may decrease cell expansion or division (Lastdrager et al. 2014) even though the existing (but fewer) cells retain proper cell wall content and lignin composition. Sufficient cell structure is maintained, but with reduced plant growth than if more energy were available from carbohydrates.

Notably, sugars increased rapidly as low R:B was transitioned to high R:B, to similar levels as plants under constant high R:B; conversely, high R:B to low R:B reduced sugars to constant low R:B levels (Figures 7 and S4). Thus, dynamic R:B application likely caused the intermediate effects on growth compared to static high R:B and low R:B (Figures 2, 3, 4), due to fluctuating carbohydrate concentrations under high or low R:B. Chen et al. (2019) found that dynamic treatments increased sucrose, but this was likely due to monochromatic effects and having hourly alternations of monochromatic R and B within a day, instead of development. Further, their times of harvest (during or after R or B application) were not defined, even though this would influence carbohydrate content, which changes during the day. Soluble sugars made via photosynthesis fulfill immediate energy demands during the day, such as responses to fluctuating light conditions (Zepeda et al. 2023). However, starch also accumulates during the day as carbon storage to be degraded into usable sucrose during night (Zepeda et al. 2023). Because starch concentrations were not affected by R:B in our study (Figures 7C,D and S5), two things can be stated. First, dynamic R:B spectra did not negatively impact plants' daily energy storage requirements in the form of starch accumulation. Second, although the direct link of soluble sugars to growth was not assessed in the present study, the accessible energy from soluble sugars was likely the primary benefactor for growth, as has been described (Apriyanto et al. 2022; Lastdrager et al. 2014; Zepeda et al. 2023).

### 4.7 Energy‐Use Efficiency, Phytochemical Production Efficiency, and Production Implications

In controlled environment agriculture, if the final product biomass is high, commercial value is often also high. However, there is also increased value for crops with longer shelf lives, higher (nutritional) quality, and more attractive pigmentation—each of these factors can also be influenced by the final phytochemical composition at harvest (Franco Lucas et al. 2021; Khoo et al. 2017; Li et al. 2017; Min et al. 2021). Here, both cultivars had greater energy‐use efficiency as the total exposure to high R:B increased (Table 1). Importantly, energy‐use efficiency is based on whole plant fresh weight accumulation per unit of input energy; however, this does not consider the energy used to produce the final phytochemical content. Thus, we also calculated the final phytochemical concentration per unit of input energy for both flavonoids and anthocyanins (e.g., mg·g^−1^ FW·kWh^−1^). Flavonoids and anthocyanins overall had more energy‐efficient production whenever there was a final phase of low R:B rather than high R:B (Tables 1 and S4). Interestingly, this indicates there are some contrasting patterns between phytochemical production efficiency and energy‐use efficiency for growth. We confirm that harnessing dynamic growth conditions is an ideal approach to strike a balance between the efficiencies of growth and phytochemical production. We present an approach with dynamic spectra that has practical applications in commercial production: a growth recipe initially utilizing high R:B, transitioning to low R:B in the final phase of cultivation. High R:B for a large part of the cultivation time can reduce electricity costs and promote efficient biomass accumulation. The end of production is ideal for growers to apply low R:B (limiting its duration and impact on biomass) to increase pigmentation and nutritional quality, as we found earlier phases of production minimally affect the final phytochemical content.

## 5 Considerations and Future Directions

One aim of this study was to define trade‐offs between growth and quality by analyzing the accumulation of select phenylpropanoids. Our study takes a novel approach by applying R:B ratios in three growth phases; then it compares how static and dynamic R:B treatments affect lettuce growth, pigments, and flavonoid production. This paper is also among the first specifically considering R:B spectra effects on lignin content, though we found limited impact. This is a small piece of the puzzle, as a limited subset of potentially involved phenylpropanoids was analyzed. There are a multitude of phenylpropanoids, as their production constitutes a complex web of metabolic pathways (Dixon and Paiva 1995; Vogt 2009). Outside of the (large) groups of flavonoids, anthocyanins, and lignin, other branches in phenylpropanoid production include esters, coumarins, phenylpropenes, and flavonols (Dong and Lin 2021; Vogt 2009). Other specialized metabolites and hormones also influence growth or are signals for metabolite production, such as auxin (Besseau et al. 2007). Therefore, the intricate and often overlapping roles of metabolites in plant growth and quality should be considered.

We also explored whether end‐of‐cultivation low R:B could improve quality with increased phytochemical content at harvest. Studies often apply changes to light spectra or intensity at the end of production (Kelly and Runkle 2023; Larsen et al. 2022; Min et al. 2021), causing difficulties in distinguishing if plant responses are due to treatments or the timing of application. By using each combination of low and high R:B in three phases, we could distinguish these potential confounding factors (e.g., confirming early low R:B phases are less impactful than the final phase). Constant low R:B reduced fresh weight compared to constant high R:B for red lettuce (44% decrease) and green lettuce (41%); however, constant low R:B also improved plant quality, increasing chlorophyll, flavonoids, and anthocyanins. Importantly, plants grown under low R:B for the final 9 days of cultivation had similar quality to plants under constant low R:B. Compared to constant high R:B, the fresh weight in these dynamic treatments was only reduced by 23% (red lettuce) or 21% (green lettuce), ~36% larger than constant low R:B. Therefore, crop quality can be improved, mitigating growth reductions, by applying low R:B near the end of production. This can likely be further improved, reducing days of low R:B exposure, or using dynamic treatments within a photoperiod, as in Van Brenk et al. (2025).

Here, we showed dynamic light treatments have significant repercussions and benefits for metabolite composition and crop growth. Future studies can expand on our use of R:B spectra. For instance, adding far‐red light (700–800 nm) to an R:B background strongly enhances plant growth, albeit with reduced metabolite content (Van Brenk et al. 2024). Notably, the final metabolite concentrations in the present study depended on the final R:B exposure. By combining far‐red in early production to induce growth, followed by a specific spectral combination to boost desired phytochemicals in late production, producers can simultaneously improve crop growth and quality (Kaiser et al. 2024). Many other factors affect plant responses (e.g., UV light, humidity, nutrient availability), which should also be further studied using dynamic growth conditions; we are just starting to scratch the surface of dynamic plant growth.

## 6 Conclusions

Growth is reduced, but metabolites are increased, with exposure to light with low red:blue ratio. In this study, we found that the impact on growth is due to the cumulative exposure to low red:blue. In contrast, we found that metabolite content is primarily determined by the last days of spectral exposure. Notably, low red:blue increased quality‐related metabolites including phenylpropanoids such as flavonoids and anthocyanins, but decreased carbohydrates and did not affect lignin content. We conclude that a growth reduction by low red:blue is therefore likely due to reduced energy from carbohydrates, rather than reduced lignin content. The regulation of these effects under different R:B spectra should be further scrutinized in future studies by performing analyses on key molecular regulators involved in light spectra response. Additionally, for metabolites affected by red:blue, the final phase of growth spectra (either high or low red:blue) had the most significant impact on final metabolite concentrations. In fact, the final 9 days of red:blue exposure exceeded the impact of all 18 days of previous growth spectra. Thus, phytochemical content mainly depends on the spectral conditions during the final growth phase, rather than preceding spectra. Therefore, using dynamic lighting, plant quality can be improved at the end of production, while earlier periods can focus on growth.

## Author Contributions

**Jordan B. Van Brenk:** conceptualization. **Jordan B. Van Brenk, Lonneke Hendriks, Andrea Rei:** methodology. **Jordan B. Van Brenk:** validation. **Jordan B. Van Brenk, Lonneke Hendriks, Andrea Rei:** formal analysis. **Jordan B. Van Brenk, Lonneke Hendriks, Andrea Rei:** investigation. **Jordan B. Van Brenk, Leo F. M. Marcelis, Julian C. Verdonk:** resources. **Jordan B. Van Brenk:** writing – original draft. **Jordan B. Van Brenk, Lonneke Hendriks, Andrea Rei, Leo F. M. Marcelis, Julian C. Verdonk:** writing – review and editing. **Jordan B. Van Brenk:** visualization. **Jordan B. Van Brenk, Leo F. M. Marcelis, Julian C. Verdonk:** supervision. **Leo F. M. Marcelis, Julian C. Verdonk:** project administration. **Leo F. M. Marcelis:** funding acquisition.

## Conflicts of Interest

The authors declare no conflicts of interest.

## Supporting information


**Figure S1:** Representative photos of cv. Redflash lettuce from the first two harvest phases.
**Figure S2:** Representative photos of cv. Greenflash lettuce from the first two harvest phases.
**Figure S3:** Specific anthocyanin concentrations of lettuce grown in this study.
**Figure S4:** Specific carbohydrate concentrations of lettuce grown in this study.
**Figure S5:** Carbohydrate composition as a percent of dry weight of lettuce grown in this study.
**Table S1:** Morphological parameters of lettuce grown in this study.
**Table S2:** Photosynthetic pigment concentrations of lettuce grown in this study.
**Table S3:** Cell wall and cell wall‐related compound concentrations of lettuce grown in this study.
**Table S4:** Phytochemical production efficiency of phytochemicals in lettuce grown in this study.

## Data Availability

The data that support the findings of this study are available from the corresponding author upon reasonable request.
