# Supplementary material for: Dynamic Application of High and Low Red:Blue Ratios During Lettuce Development Shifts Growth and Metabolite Allocation
Source: Physiol Plant. 2025 Aug 13;177(4):e70456. doi: 10.1111/ppl.70456 (PMC12351208; doi:10.1111/ppl.70456)
Supplement: Supplementary file 1 — Figure S1: Representative photos of cv. Redflash lettuce from the first two harvest phases. Figure S2: Representative photos of cv. Greenflash lettuce from the first two harvest phases. Figure S3: Specific anthocyanin concentrations of lettuce grown in this study. Figure S4: Specific carbohydrate concentrations of lettuce grown in this study. Figure S5: Carbohydrate composition as a percent of dry weight of lettuce grown in this study. Table S1: Morphological parameters of lettuce grown in this study. Table S2: Photosynthetic pigment concentrations of lettuce grown in this study. Table S3: Cell wall and cell wall‐related compound concentrations of lettuce grown in this study. Table S4: Phytochemical production efficiency of phytochemicals in lettuce grown in this study. [file PPL-177-e70456-s001.pdf]

Supporting information for:

Dynamic application of high and low red:blue ratios during lettuce development shifts growth and metabolite allocation

Jordan B Van Brenk[1], Lonneke Hendriks[1], Andrea Rei[1], Leo FM Marcelis[1], Julian C Verdonk[*1]

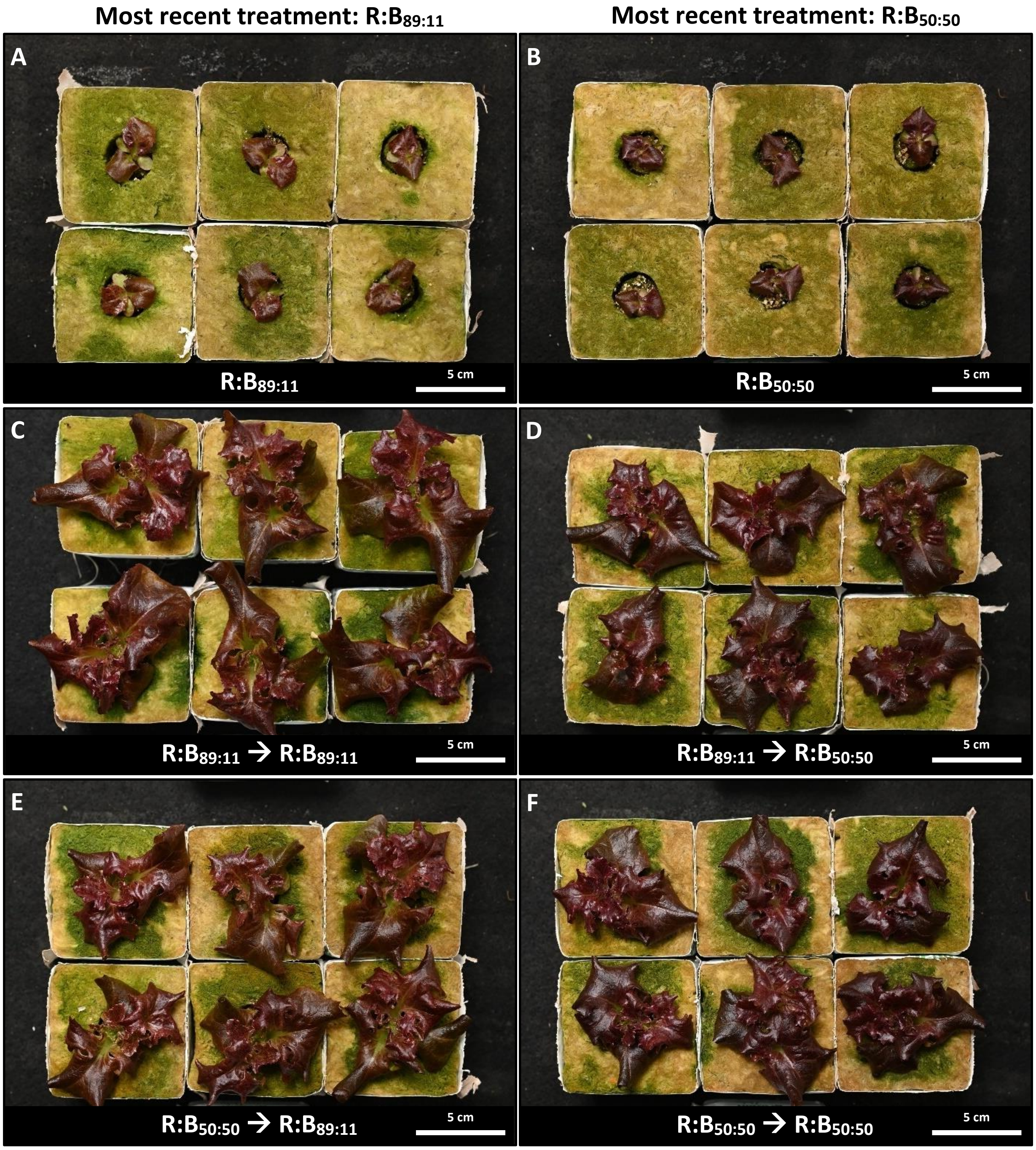

Supplemental Figure S1. Representative overhead photos of cv. Redflash lettuce grown under high or low red:blue ratio for one or two nine-day phases

Representative visual appearance (overhead photos) of cv. Redflash at 9 (**A**, **B**) or 18 (**C-F**) days after transplant (DAT), displaying the effects of red:blue photon ratios applied during phase 1 (0-9 DAT) and phase 2 (9-18 DAT). Two different R:B ratios (R:$B_{89:11}$ and R:$B_{50:50}$) were applied during these nine-day phases of growth; denoted at the bottom of each photograph is the red:blue ratio used in each phase of plant growth (Phase 1 → Phase 2).

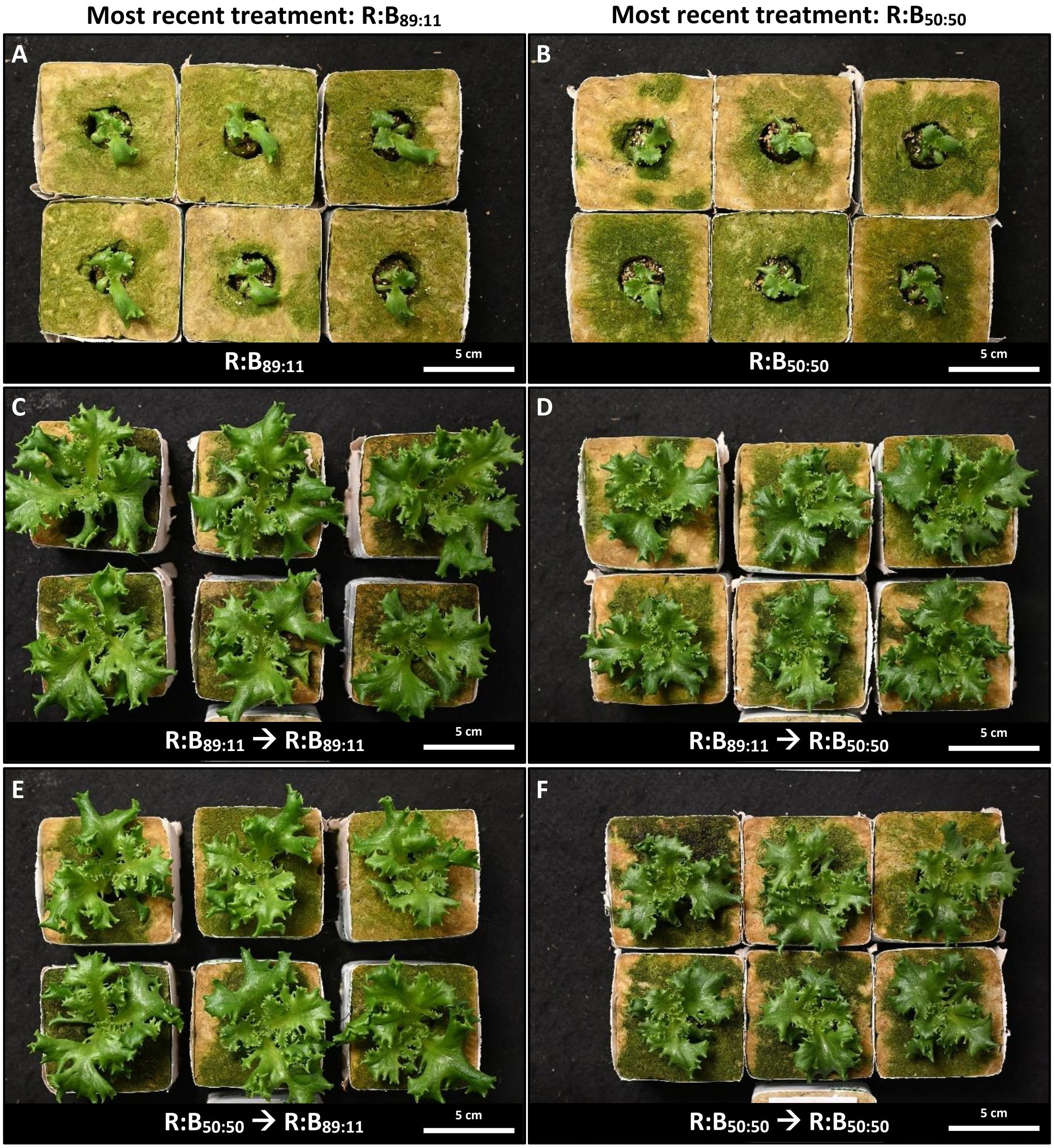

Supplemental Figure S2. Representative overhead photos of cv. Greenflash lettuce grown under high or low red:blue ratio for one or two nine-day phases

Representative visual appearance (overhead photos) of cv. Greenflash at 9 (**A**, **B**) or 18 (**C-F**) days after transplant (DAT), displaying the effects of red:blue photon ratios applied during phase 1 (0-9 DAT) and phase 2 (9-18 DAT). Two different R:B ratios ($R:B_{89:11}$ and $R:B_{50:50}$) were applied during these nine-day phases of growth; denoted at the bottom of each photograph is the red:blue ratio used in each phase of plant growth (Phase 1 → Phase 2).

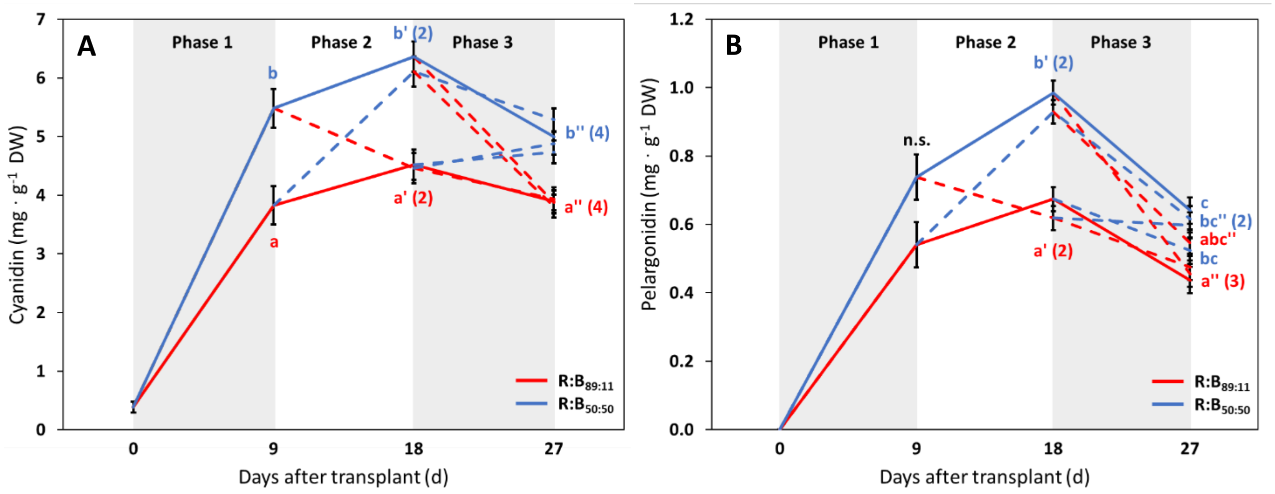

Supplemental Figure S3. Effects on cyanidin and pelargonidin from nine-day periods of high or low red:blue ratio during three different phases of cultivation for a red lettuce cultivar

Cyanidin concentration (**A**; mg · g^{-1}) and pelargonidin concentration (**B**; mg · g^{-1}) in cv. Redflash grown for a total of 27 days after transplant, with three nine-day phases of receiving either $R:B_{89:11}$ (red line) or $R:B_{50:50}$ (blue line). Solid lines represent constant treatments of $R:B_{89:11}$ or $R:B_{50:50}$ for the whole growth cycle, dashed lines represent treatments that transition between the two R:B ratios. Datapoints represent means with standard error means of four growth cycles (n = 4), each consisting of six replicate plants. A one-way ANOVA was performed within each phase. Different letters indicate significantly different values for each treatment within a phase, according to a protected Fisher LSD test (a = 0.05), for Phase 1 (no apostrophe), Phase 2 ('), and Phase 3 (").

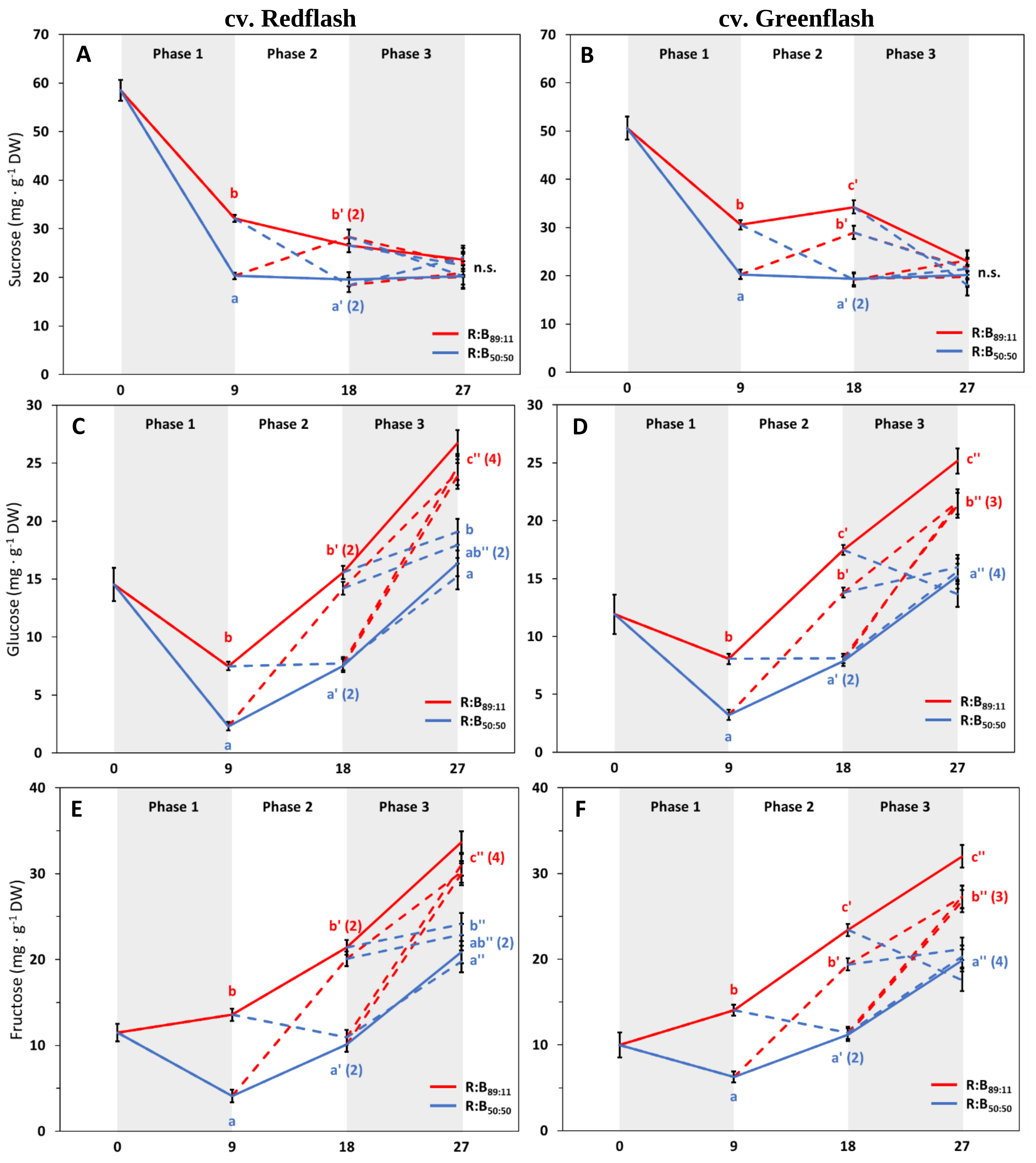

Supplemental Figure S4. Effects on soluble carbohydrates from nine-day periods of high or low red:blue ratio during three different phases of cultivation for two lettuce cultivars

Sucrose (**A**, **B**; mg · g^{-1}), glucose (**C**, **D**; mg · g^{-1}), and fructose (**E**, **F**; mg · g^{-1}) in cv. Redflash (**A**, **C**, **E**) and cv. Greenflash (**B**, **D**, **F**) grown for a total of 27 days after transplant, with three nine-day phases of receiving either $R{:}B_{89:11}$ (red line) or $R{:}B_{50:50}$ (blue line). Solid lines represent constant treatments of $R{:}B_{89:11}$ or $R{:}B_{50:50}$ for the whole growth cycle, dashed lines represent treatments that transition between the two R:B ratios. Datapoints represent means with standard error means of four growth cycles ($n = 4$), each consisting of six replicate plants. A one-way ANOVA was performed within each

phase. Different letters indicate significantly different values for each treatment within a phase, according to a protected Fisher LSD test (*a* = 0.05), for Phase 1 (no apostrophe), Phase 2 ('), and Phase 3 ('').

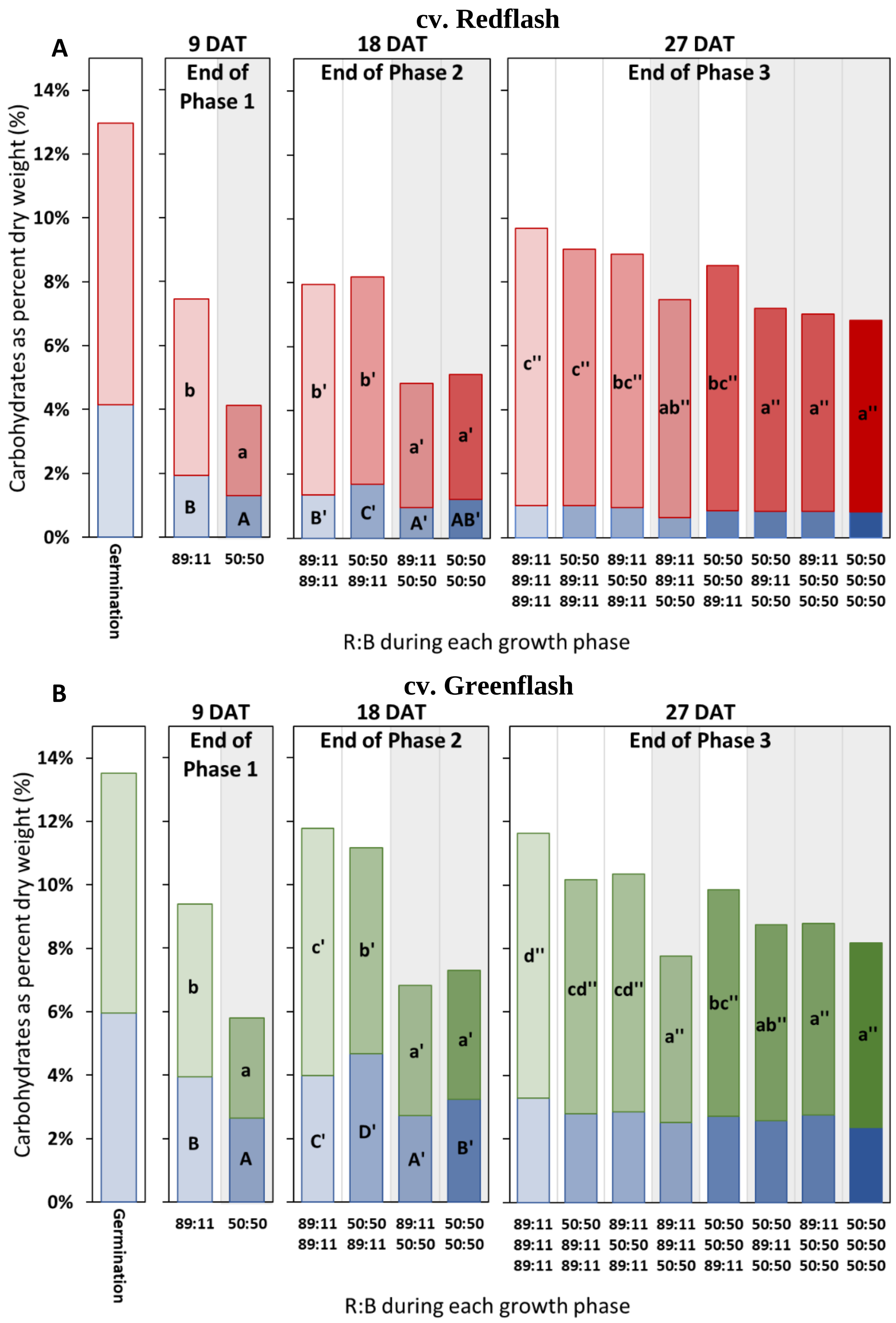

Supplemental Figure S5. Effects on total soluble sugars and starch as a percentage of dry weight from nine-day periods of high or low red:blue ratio during three different phases of cultivation for two lettuce cultivars

Total soluble sugars (red bars in **A** and green in **B**) and starch (blue bars) as a percent of dry weight (%) of lettuce cv. Redflash (**A-C**) and cv. Greenflash (**D-F**) grown for up to 27 days after transplant. Two different R:B ratios ($R{:}B_{89:11}$ and $R{:}B_{50:50}$) were applied during each of three nine-day phases of growth (Phase 1, Phase 2, and Phase 3). Datapoints represent means of four growth cycles (n = 4), each

consisting of six replicate plants. A one-way ANOVA was performed within each phase. Different letters (lowercase for soluble sugars, uppercase for starch) indicate significantly different values for each treatment within a phase, according to a protected Fisher LSD test (*a* = 0.05), for Phase 1 (no apostrophe), Phase 2 ('), and Phase 3 ("). The colour and order of data bars correspond with the number of phases that low R:B was applied, with a grey background indicating a final phase of low R:B.

Supplemental Table S1. Effects on morphological parameters from nine-day periods of high or low red:blue ratio during three different phases of cultivation for two lettuce cultivars

	DAT[*]	R:B[**] during days: 0-9 (Phase 1)	10-18 (Phase 2)	19-27 (Phase 3)	Dry weight (g)	Leaf area (cm^2)	Leaf number	Specific leaf area ($cm^2 \cdot g^{-1}$ DW)	Dry matter content (%)
cv. Redflash	0	--[***]	--	--	0.0014	0.64	0	455	8.49
	9	89:11	--	--	0.019^{b}	8.13^{b}	2.0	416	7.97
	9	50:50	--	--	0.015^{a}	6.09^{a}	2.1	399	8.10
	18	89:11	89:11	--	0.273$^{c'}$	110.5$^{c'}$	5.4$^{b'}$	407	6.65
	18	50:50	89:11	--	0.219$^{b'}$	86.5$^{b'}$	5.3$^{b'}$	395	6.74
	18	89:11	50:50	--	0.167$^{a'}$	66.6$^{a'}$	5.0$^{a'}$	398	6.69
	18	50:50	50:50	--	0.162$^{a'}$	63.3$^{a'}$	5.3$^{b'}$	389	6.73
	27	89:11	89:11	89:11	1.361$^{e''}$	563$^{d''}$	10.9$^{d''}$	415	6.30
	27	50:50	89:11	89:11	1.262$^{e''}$	504$^{d''}$	10.5$^{cd''}$	401	6.48
	27	89:11	50:50	89:11	1.088$^{d''}$	432$^{c''}$	10.2$^{bc''}$	398	6.45
	27	50:50	50:50	89:11	1.056$^{cd''}$	429$^{c''}$	10.3$^{bc''}$	406	6.42
	27	89:11	89:11	50:50	1.066$^{cd''}$	434$^{c''}$	10.1$^{bc''}$	408	6.45
	27	50:50	89:11	50:50	0.953$^{bc''}$	375$^{bc''}$	10.0$^{abc''}$	395	6.54
	27	89:11	50:50	50:50	0.885$^{ab''}$	341$^{ab''}$	9.9$^{ab''}$	385	6.52
	27	50:50	50:50	50:50	0.775$^{a''}$	297$^{a''}$	9.5$^{a''}$	383	6.52
	**SEM for:**		9 DAT		0.0013	0.26	0.0589	8.07	0.077
			18 DAT'		0.0081'	2.85'	0.1002'	8.08'	0.035'
			27 DAT''		0.0452''	20.47''	0.1947''	8.83''	0.099''
	***$P_{Treatment}$* for:**		9 DAT		**0.041**	**0.011**	0.391	0.238	0.308
			18 DAT'		**<0.001'**	**<0.001'**	**0.045'**	0.244'	0.283'
			27 DAT''		**<0.001''**	**<0.001''**	**0.003''**	0.205''	0.759''
cv. Greenflash	0	--	--	--	0.0018	0.44	0	258	12.54
	9	89:11	--	--	0.019^{b}	6.14^{b}	2.7	328	7.69
	9	50:50	--	--	0.015^{a}	4.39^{a}	2.8	299	8.08
	18	89:11	89:11	--	0.222$^{c'}$	70.8$^{b'}$	7.0	319	7.23
	18	50:50	89:11	--	0.199$^{bc'}$	62.2$^{b'}$	6.9	312	7.41
	18	89:11	50:50	--	0.169$^{ab'}$	50.7$^{a'}$	6.5	301	7.37
	18	50:50	50:50	--	0.160^{a}	46.4$^{a'}$	7.1	292	7.31
	27	89:11	89:11	89:11	1.345$^{e''}$	474$^{f''}$	14.3	352$^{d''}$	6.65$^{a''}$
	27	50:50	89:11	89:11	1.272$^{de''}$	436$^{ef''}$	13.7	342$^{cd''}$	6.91$^{ab''}$
	27	89:11	50:50	89:11	1.046$^{b''}$	342$^{bcd''}$	13.2	327$^{bc''}$	6.88$^{ab''}$
	27	50:50	50:50	89:11	1.010$^{bc''}$	360$^{cd''}$	13.5	355$^{d''}$	6.82$^{ab''}$
	27	89:11	89:11	50:50	1.158$^{cd''}$	383$^{de''}$	13.7	330$^{c''}$	7.11$^{bc''}$
	27	50:50	89:11	50:50	1.029$^{bc''}$	308$^{abc''}$	13.2	301$^{a''}$	7.25$^{c''}$
	27	89:11	50:50	50:50	0.898$^{ab''}$	297$^{ab''}$	13.2	331$^{c''}$	7.05$^{bc''}$
	27	50:50	50:50	50:50	0.843$^{a''}$	262$^{a''}$	12.9	309$^{ab''}$	7.06$^{bc''}$
	**SEM for:**		9 DAT		0.0004	0.08	0.0589	8.24	0.113
			18 DAT'		0.0102'	3.48'	0.1889'	6.63'	0.053'
			27 DAT''		0.0525''	20.62''	0.2672''	6.54''	0.101''
	***$P_{Treatment}$* for:**		9 DAT		**0.007**	**<0.001**	0.391	0.084	0.093
			18 DAT'		**0.007'**	**0.003'**	0.223'	0.072'	0.135'
			27 DAT''		**<0.001''**	**<0.001''**	0.094''	**<0.001''**	**0.012''**

*DAT = Days after transplant.
**The indicated R:B was applied during three phases that lasted nine days.
***-- indicates R:B treatment not yet applied
Note: Data are means of four growth cycles (*n* = 4), each consisting of six replicate plants. A one-way ANOVA was performed within each phase. Different letters indicate significantly different values for each treatment within a phase, according to a protected Fisher LSD test (*a* = 0.05), for 9 DAT/Phase 1 (no apostrophe), 18 DAT/Phase 2 ('), and 27 DAT/Phase 3 ("). SEM = standard error of means. $P_{Treatment}$ = probability of an effect from R:B treatments.

Supplemental Table S2. Effects on photosynthetic pigments from nine-day periods of high or low red:blue ratio during three different phases of cultivation for two lettuce cultivars

	DAT[*]	R:B[**] during days: 0-9 (Phase 1)	10-18 (Phase 2)	19-27 (Phase 3)	Chlorophyll A (mg · g^{-1} DW)	Chlorophyll B (mg · g^{-1} DW)	Chl A/B ratio	Carotenoids (mg · g^{-1} DW)
cv. Redflash	0	--[***]	--	--	2.189	1.110	1.970	0.3818
	9	89:11	--	--	2.980[a]	1.261[a]	2.358	0.6142
	9	50:50	--	--	3.570[b]	1.584[b]	2.247	0.6219
	18	89:11	89:11	--	4.234[a']	1.785[a']	2.373	0.7669
	18	50:50	89:11	--	4.154[a']	1.745[a']	2.382	0.7548
	18	89:11	50:50	--	4.845[b']	2.070[b']	2.341	0.7993
	18	50:50	50:50	--	5.061[c']	2.196[c']	2.307	0.8145
	27	89:11	89:11	89:11	4.302[a'']	1.840[a'']	2.338	0.7593
	27	50:50	89:11	89:11	4.381[a'']	1.841[a'']	2.383	0.7827
	27	89:11	50:50	89:11	4.383[a'']	1.824[a'']	2.403	0.7698
	27	50:50	50:50	89:11	4.418[ab'']	1.833[a'']	2.410	0.7964
	27	89:11	89:11	50:50	4.746[bc'']	2.017[bc'']	2.348	0.7801
	27	50:50	89:11	50:50	4.645[ab'']	1.971[ab'']	2.358	0.7877
	27	89:11	50:50	50:50	5.060[cd'']	2.148[cd'']	2.357	0.8269
	27	50:50	50:50	50:50	5.136[d'']	2.185[d'']	2.350	0.8308
	**SEM for:**			9 DAT	0.1009	0.0207	0.0491	0.0248
				18 DAT'	0.0577'	0.0278'	0.0326'	0.0175'
				27 DAT''	0.1171''	0.0515''	0.0205''	0.0263''
	***$P_{Treatment}$* for:**			9 DAT	**0.026**	**0.002**	0.207	0.840
				18 DAT'	**<0.001'**	**<0.001'**	0.402'	0.124'
				27 DAT''	**<0.001''**	**<0.001''**	0.153''	0.499''
cv. Greenflash	0	--	--	--	4.485	2.304	1.923	0.724
	9	89:11	--	--	5.410	2.633	2.053	0.878
	9	50:50	--	--	5.731	2.874	1.989	0.854
	18	89:11	89:11	--	7.133	3.386[a']	2.106	1.179
	18	50:50	89:11	--	7.301	3.437[ab']	2.124	1.185
	18	89:11	50:50	--	7.760	3.715[c']	2.086	1.211
	18	50:50	50:50	--	7.629	3.660[bc']	2.083	1.202
	27	89:11	89:11	89:11	7.706[ab'']	3.623[ab'']	2.125	1.291
	27	50:50	89:11	89:11	7.547[a'']	3.509[a'']	2.150	1.318
	27	89:11	50:50	89:11	8.235[abc'']	3.826[abc'']	2.148	1.388
	27	50:50	50:50	89:11	7.690[ab'']	3.594[ab'']	2.141	1.308
	27	89:11	89:11	50:50	8.698[c'']	4.118[c'']	2.111	1.404
	27	50:50	89:11	50:50	8.623[bc'']	4.027[bc'']	2.143	1.410
	27	89:11	50:50	50:50	8.992[c'']	4.220[c'']	2.132	1.398
	27	50:50	50:50	50:50	8.359[abc'']	3.943[abc'']	2.121	1.359
	**SEM for:**			9 DAT	0.2500	0.1206	0.0289	0.0385
				18 DAT'	0.1813'	0.0722'	0.0132'	0.0241'
				27 DAT''	0.3340''	0.1511''	0.0215''	0.0526''
	***$P_{Treatment}$* for:**			9 DAT	0.430	0.254	0.214	0.694
				18 DAT'	0.121'	**0.025'**	0.185'	0.766'
				27 DAT''	**0.045''**	**0.023''**	0.879''	0.575''

*DAT = Days after transplant.
**The indicated R:B was applied during three phases that lasted nine days.
***-- indicates R:B treatment not yet applied
Note: Data are means of four growth cycles ($n = 4$), each consisting of a pooled sample of six replicate plants. A one-way ANOVA was performed within each phase. Different letters indicate significantly different values for each treatment within a phase, according to a protected Fisher LSD test ($a = 0.05$), for 9 DAT/Phase 1 (no apostrophe), 18 DAT/Phase 2 ('), and 27 DAT/Phase 3 ("). SEM = standard error of means. $P_{Treatment}$ = probability of an effect from R:B treatments.

Supplemental Table S3. Effects on cell wall, lignin, caffeic acid, and chlorogenic acid content from nine-day periods of high or low red:blue ratio during three different phases of cultivation for two lettuce cultivars

	DAT[*]	R:B[**] during days: 0-9 (Phase 1)	10-18 (Phase 2)	19-27 (Phase 3)	Cell wall content (% DW)	G monomer (relative)	S monomer (relative)	S/G ratio	Caffeic acid (mg · g^{-1} DW)	Chlorogenic acid (mg · g^{-1} DW)
cv. Redflash	0	--[***]	--	--	45.3	0.1648	0.0752	0.432	11.91	29.90
	9	89:11	--	--	55.8	0.1180	0.0529	0.414	12.76	30.27
	9	50:50	--	--	60.9	0.0990	0.0431	0.414	13.21	32.81
	18	89:11	89:11	--	51.9	0.1489	0.0690	0.413	13.42	24.03
	18	50:50	89:11	--	52.6	0.1548	0.0690	0.442	12.13	25.11
	18	89:11	50:50	--	60.1	0.1355	0.0481	0.379	14.35	27.60
	18	50:50	50:50	--	51.8	0.1587	0.0619	0.358	14.19	25.93
	27	89:11	89:11	89:11	$51.3^{bc''}$	0.1973	0.0540	0.245	12.09	23.84
	27	50:50	89:11	89:11	$56.0^{d''}$	0.1722	0.0541	0.304	12.97	25.45
	27	89:11	50:50	89:11	$54.6^{cd''}$	0.1820	0.0407	0.230	12.48	24.15
	27	50:50	50:50	89:11	$49.5^{ab''}$	0.2539	0.0567	0.207	12.18	25.49
	27	89:11	89:11	50:50	$51.2^{bc''}$	0.2336	0.0655	0.288	12.68	25.82
	27	50:50	89:11	50:50	$48.2^{ab''}$	0.2559	0.1074	0.387	13.39	26.04
	27	89:11	50:50	50:50	$57.4^{d''}$	0.2681	0.0732	0.248	13.31	27.05
	27	50:50	50:50	50:50	$45.9^{a''}$	0.2055	0.0573	0.278	13.53	26.32
	**SEM for:**			9 DAT	3.18	0.0159	0.01339	0.0640	0.379	1.880
				18 DAT'	2.18'	0.0283'	0.01609'	0.0523'	0.581'	1.191'
				27 DAT''	1.49''	0.0387''	0.01751''	0.0376''	0.561''	0.846''
	**$P_{Treatment}$ for:**			9 DAT	0.341	0.464	0.639	0.999	0.461	0.409
				18 DAT'	0.069'	0.941'	0.773'	0.689'	0.086'	0.257'
				27 DAT''	**<0.001''**	0.529''	0.294''	0.076''	0.470''	0.187''
cv. Greenflash	0	--	--	--	54.5	0.0972	0.0565	0.541	9.126	33.89
	9	89:11	--	--	52.1	0.0721	0.0334	0.445	6.172	31.51^{a}
	9	50:50	--	--	60.2	0.0811	0.0435	0.531	7.418	34.19^{b}
	18	89:11	89:11	--	50.1	0.1449	0.0599	0.401	5.962	25.76
	18	50:50	89:11	--	53.6	0.0794	0.0322	0.399	5.934	28.22
	18	89:11	50:50	--	56.3	0.1270	0.0532	0.430	6.258	27.94
	18	50:50	50:50	--	58.2	0.1037	0.0498	0.434	6.162	27.57
	27	89:11	89:11	89:11	$46.8^{ab''}$	0.1476	0.0499	0.299	5.736	29.79
	27	50:50	89:11	89:11	$51.6^{bc''}$	0.1824	0.0678	0.327	5.966	31.69
	27	89:11	50:50	89:11	$55.9^{cd''}$	0.1542	0.0587	0.346	5.494	31.71
	27	50:50	50:50	89:11	$45.8^{a''}$	0.1723	0.0686	0.349	5.849	29.67
	27	89:11	89:11	50:50	$52.1^{c''}$	0.1664	0.0544	0.321	5.332	31.57
	27	50:50	89:11	50:50	$44.7^{a''}$	0.1748	0.0550	0.309	6.489	33.07
	27	89:11	50:50	50:50	$59.8^{d''}$	0.1191	0.0362	0.281	5.443	32.82
	27	50:50	50:50	50:50	$42.2^{a''}$	0.1632	0.0515	0.318	5.684	30.32
	**SEM for:**			9 DAT	5.87	0.0088	0.00565	0.0420	0.514	0.503
				18 DAT'	2.29'	0.0220'	0.01198'	0.0375'	0.334'	0.590'
				27 DAT''	01.75''	0.0276''	0.01212''	0.0346''	0.329''	1.241''
	**$P_{Treatment}$ for:**			9 DAT	0.403	0.518	0.264	0.264	0.185	**0.033**
				18 DAT'	0.144'	0.246'	0.448'	0.863'	0.881'	0.061'
				27 DAT''	**<0.001''**	0.806''	0.646''	0.867''	0.325''	0.406''

*DAT = Days after transplant.
**The indicated R:B was applied during three phases that lasted nine days.
***-- indicates R:B treatment not yet applied
Note: Data are means of four growth cycles (*n* = 4), each consisting of a pooled sample of six replicate plants. A one-way ANOVA was performed within each phase. Different letters indicate significantly different values for each treatment within a phase, according to a protected Fisher LSD test (a = 0.05), for 9 DAT/Phase 1 (no apostrophe), 18 DAT/Phase 2 ('), and 27 DAT/Phase 3 ("). SEM = standard error of means. $P_{Treatment}$ = probability of an effect from R:B treatments.

Supplemental Table S4. Phytochemical production efficiency per gram dry weight and per plant grown under nine-day periods of high or low red:blue ratio during three different phases of cultivation for two lettuce cultivars

	R:B* during days:			Flavonoids		Anthocyanins**	
	**0-9** (Phase 1)	**10-18** (Phase 2)	**19-27** (Phase 3)	$\left(\frac{\text{mg}/g\ DW}{\text{kWh}}\right)$	$\left(\frac{\text{mg}/plant}{\text{kWh}}\right)$	$\left(\frac{A/g\ DW}{\text{kWh}}\right)$	$\left(\frac{A/plant}{\text{kWh}}\right)$
cv. Redflash at 27 DAT	89:11	89:11	89:11	40.8	55.5^{d}	2537^{a}	3443^{d}
	50:50	89:11	89:11	42.1	52.9^{d}	2572^{a}	3248^{d}
	89:11	50:50	89:11	40.3	44.3^{bc}	2522^{a}	2727^{bc}
	50:50	50:50	89:11	43.2	45.9^{c}	2383^{a}	2528^{ab}
	89:11	89:11	50:50	44.0	46.7^{c}	2880^{b}	3069^{cd}
	50:50	89:11	50:50	45.6	43.3^{bc}	2917^{b}	2786^{bc}
	89:11	50:50	50:50	43.8	38.7^{ab}	3096^{b}	2697^{bc}
	50:50	50:50	50:50	44.4	34.8^{a}	2856^{b}	2235^{a}
	**SEM**			1.43	1.98	94.9	147.2
	$\boldsymbol{P_{Treatment}}$			0.175	**<0.001**	**<0.001**	**<0.001**
cv. Greenflash at 27 DAT	89:11	89:11	89:11	46.5^{ab}	62.2^{cd}	N/A	N/A
	50:50	89:11	89:11	44.4^{a}	56.4^{c}	N/A	N/A
	89:11	50:50	89:11	52.3^{bc}	54.7^{bc}	N/A	N/A
	50:50	50:50	89:11	46.3^{ab}	47.0^{ab}	N/A	N/A
	89:11	89:11	50:50	59.0^{de}	67.2^{d}	N/A	N/A
	50:50	89:11	50:50	62.3^{e}	63.8^{cd}	N/A	N/A
	89:11	50:50	50:50	63.7^{e}	57.1^{c}	N/A	N/A
	50:50	50:50	50:50	53.1^{cd}	44.5^{a}	N/A	N/A
	**SEM**			2.15	3.21	N/A	N/A
	$\boldsymbol{P_{Treatment}}$			**<0.001**	**<0.001**	N/A	N/A

*The indicated R:B was applied during three phases that lasted nine days.

**For anthocyanins, relative absorbance (A) was calculated as A_{530}-A_{657}.

Abbreviations: R:B = red:blue light ratio, DW = dry weight, kWh = kilowatt-hour, DAT = days after transplant, N/A = not applicable.

Note: Data are means of four growth cycles (n = 4), each consisting of six replicate plants. A one-way ANOVA was performed. Different letters indicate significantly different values for each treatment within a phase, according to a protected Fisher LSD test (a = 0.05). SEM = standard error of means. $P_{Treatment}$ = probability of an effect from R:B treatments.
